# Supplementary material for: Assessing the Orthogonality of Phage-Encoded RNA Polymerases for Tailored Synthetic Biology Applications in Pseudomonas Species
Source: Int J Mol Sci. 2023 Apr 13;24(8):7175. doi: 10.3390/ijms24087175 (PMC10138996; doi:10.3390/ijms24087175)
Supplement: Supplementary file 1 [file ijms-24-07175-s001.zip › ijms-2279831-supplementary.pdf]

Supporting information

Supplementary Experiments

Phage promoters and their native 5′UTR are co-evolved to yield high expression levels

To validate the expression levels of the phage RNAPs and promoters in combination with BCD2, all phage promoters were connected to BCD2 by a GGGCAG linker and cloned together with msfGFP into pBGDes. The GGGCAG linker contains the GCAG position tag required for SEVAtile shuffling and a double G directly following the TSS of the promoter. This is known to be important for proper transcription initiation of the T7 promoter (Figure 5) [21,22]. The resulting vectors were introduced together with the corresponding phage RNAP in *P. putida* KT2440.

Unexpectedly, the replacement of the MCP 5′UTR by BCD2 resulted in a significant drop in msfGFP fluorescence output for all phage RNAPs except for P_{phi15} ($P<0.05$) (Figure S1). For Pf-10 and 67PfluR64PP, almost no fluorescent intensity was detected at all, indicating that the confirmed phage promoter is not sufficient for efficient transcription by these phage polymerases. A first hypothesis for this observation led us to the unwinding region of the phage promoters. As indicated in Figure 4, the last four nucleotides of the promoters are predicted to play an important role in DNA unwinding [22]. Interestingly, this region contains three A/T nucleotides for both the Pf-10 and 67PfluR64PP promoter, while the other promoters have four A/T nucleotides. Therefore, it is reasonable to assume that four consecutive A/T nucleotides are required for proper DNA unwinding and transcription initiation of these promoters, as was confirmed for the T7 promoter in previous research [18,22].

To test this hypothesis, the GGGCAG linker from the previous construct was replaced by the two first nucleotides of the corresponding phage MCP 5′UTR followed by GCAG. In this way, all phage promoter-UTR constructs contain four consecutive A/T nucleotides, which will potentially increase the fluorescent output. This trend could indeed be observed for Pf-10 and 67PfluR64PP, but the msfGFP levels still remain about fourfold lower than the levels observed for the full MCP 5′UTR (Figure S1). For T7, phi15 and PPPL-1, no significant difference in msfGFP output was observed between constructs containing the GGGCAG linker or NNGCAG linker. These results indicate that an intact unwinding region of four consecutive A/T nucleotides is important for T7-like promoters, but does not fully explain the significant difference in fluorescence intensity between the MCP 5′UTR and BCD2 for PPPL-1, Pf-10 and 67PfluR64PP (Figure 5).

When analyzing the MCP 5′UTR further, it can be observed that all T7-like phage promoter regions in this paper contain a 13-nt stretch without any thymidine residue directly downstream of the TSS. The conservation of this T-less stretch in diverse T7-like phages could indicate that this region is important for efficient transcription by the phage RNAP. Therefore, the previous linkers are now extended with the thirteen first nucleotides of the corresponding phage's MCP 5′UTR to include the T-less stretch. The addition of the T-less stretch has a marked influence on the msfGFP expression levels of Pf-10 and 67PfluR64PP, while the effect on T7, phi15 and PPPL-

1 is much less pronounced (Figure 5**Error! Reference source not found.**). In case of 67PfluR64PP, the fluorescence intensity is sevenfold higher compared to the N_{13}-GCAG-BCD2 UTR and almost twice as high as the MCP 5′UTR. However, for all other phages the MCP 5′UTR still outperforms all UTRs containing BCD2. These results once again highlight that the phage promoter and MCP 5′UTR have been evolutionarily optimized to generate high levels of transcription together and that splitting these two parts to used them separately in synthetic circuitry is not straightforward [27].

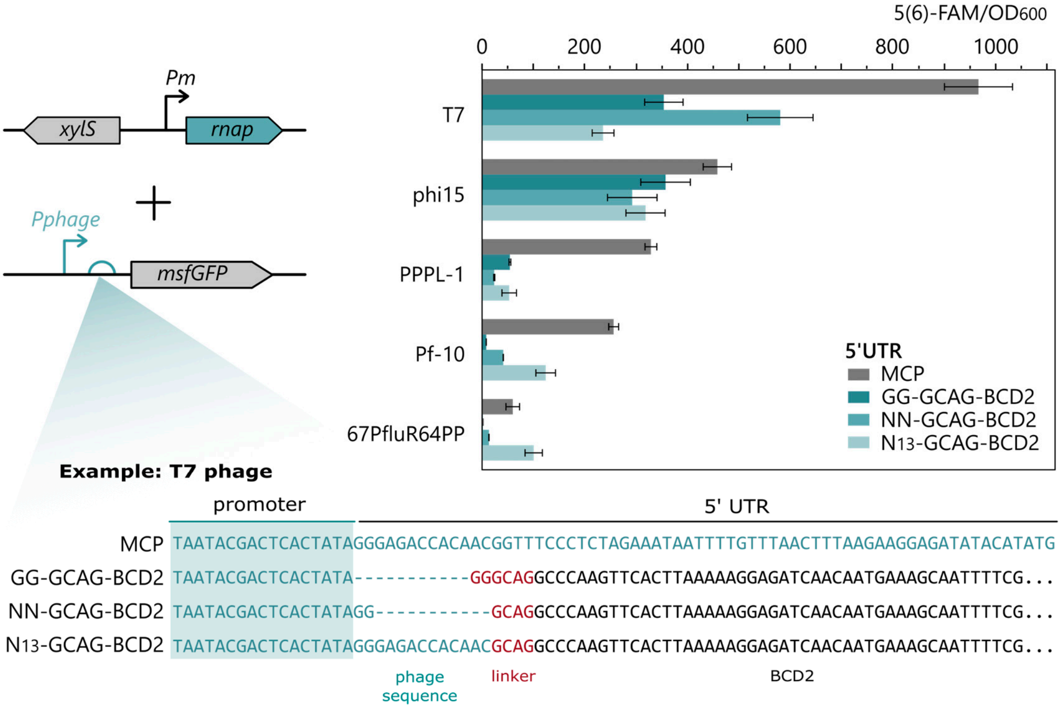

Figure S1. The sequence downstream of T7-like phage promoters highly impact transcription efficiency. For T7-like phages T7, phi15, PPPL-1, Pf-10 and 67PfluR64PP, their confirmed promoter was paired with different 5′UTRs: 1) the full 5′UTR of the corresponding phage's major capsid protein (MCP), 2) the standardized UTR BCD2, linked to the promoter by GGGCAG, 3) BCD2, linked to the promoter by the first two nucleotides of the corresponding phage's MCP and GCAG, 4) BCD2, linked to the promoter by the first thirteen nucleotides of the corresponding phage's MCP and GCAG. The combinations were cloned into pBGDes and introduced in P. putida KT2440 together with pSTDes3 carrying the corresponding phage RNAP. Bars represent the mean fluorescent intensity of four biological replicates after 6h of induction with 0.3 3mBz, expressed as equivalent 5(6)-FAM concentration and normalized for OD_{600}. Error bars represent the standard error.

Supplementary Figures

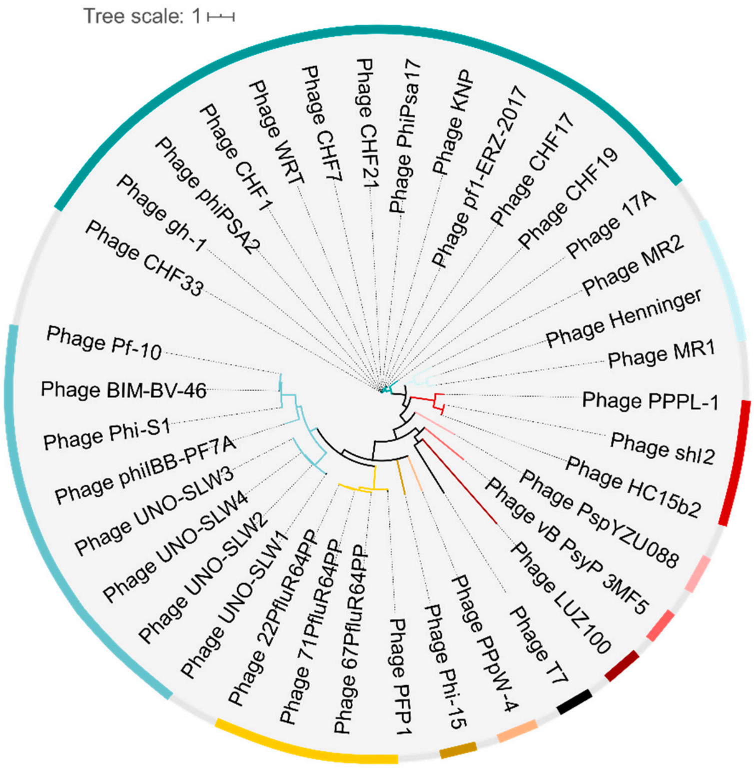

Figure S2. Phylogenetic tree (ClustalOmega) of T7-like Pseudomonas phages and coliphage T7, based on the RNAP sequence. Based on this tree, the phages can be subdivided into eleven different clades, indicated by different colors. The tree scale represents the phylogenetic distance between sequences.

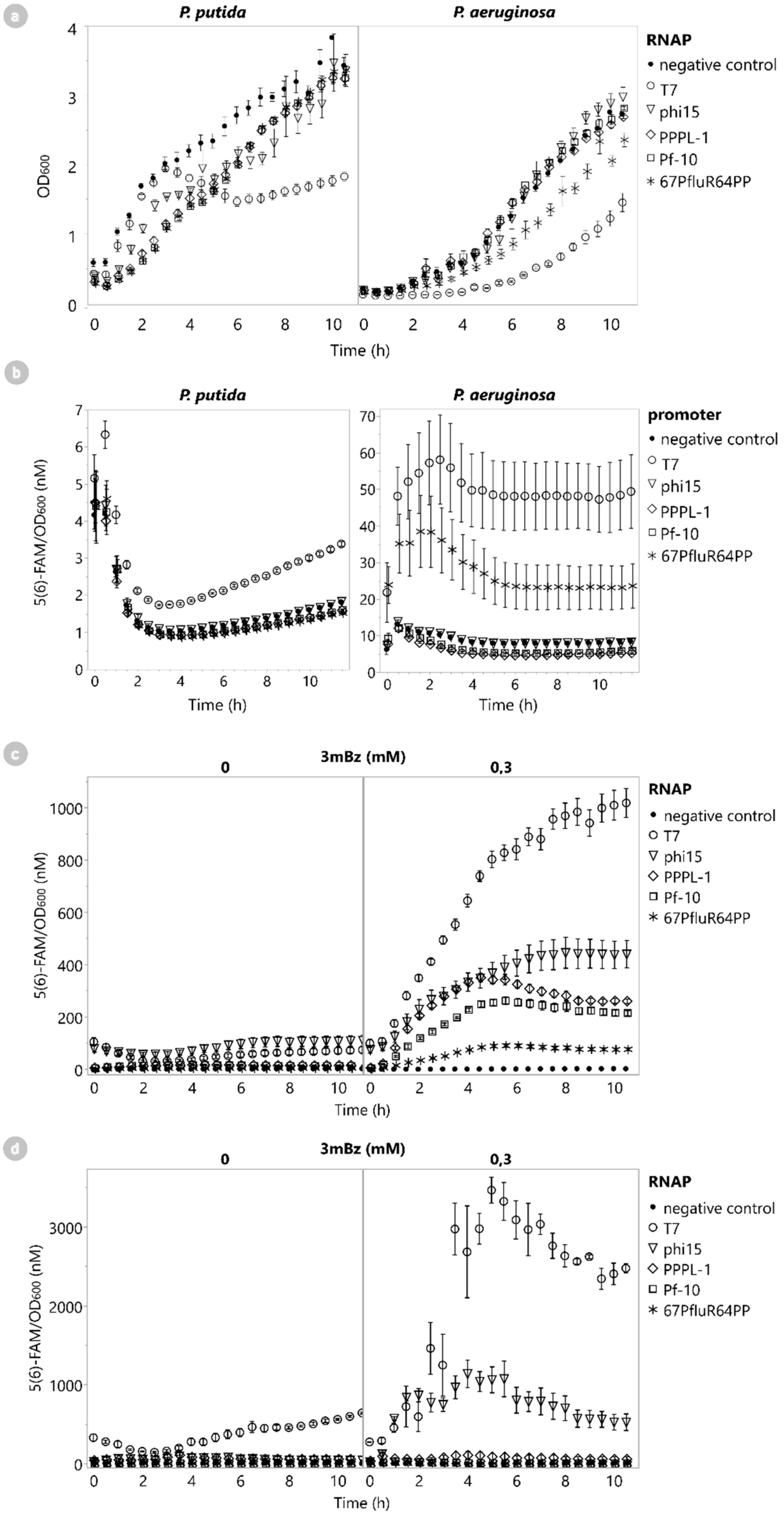

a
P. putida
P. aeruginosa
OD600
Time (h)
RNAP
negative control
T7
phi15
PPPL-1
Pf-10
67PfluR64PP
b
P. putida
P. aeruginosa
5(6)-FAM/OD600 (nM)
Time (h)
promoter
negative control
T7
phi15
PPPL-1
Pf-10
67PfluR64PP
c
3mBz (mM)
0
0,3
5(6)-FAM/OD600 (nM)
Time (h)
RNAP
negative control
T7
phi15
PPPL-1
Pf-10
67PfluR64PP
d
3mBz (mM)
0
0,3
5(6)-FAM/OD600 (nM)
Time (h)
RNAP
negative control
T7
phi15
PPPL-1
Pf-10
67PfluR64PP

Figure S3. (**a**) Effect of phage RNAP expression on cell growth of P. putida KT2440 and P. aeruginosa PAO1. All P. putida and P. aeruginosa strains RX (negative control), RA0 (T7), RB0 (phi15), RC0 (PPPL-1), RD0 (Pf-10) and RE0 (67PfluR64PP) were induced with 1 mM 3mBz, after which the OD_{600} was measured every 15 minutes for 12 hours. Markers and error bars represent the mean value and standard error of four biological replicates every 30 minutes. (**b**) Recognition of phage promoter by host RNAP of P. putida and P. aeruginosa. All P. putida and P. aeruginosa strains pX (negative control), pA0 (T7), pB0 (phi15), pC0 (PPPL-1), pD0 (Pf-10) and pE0 (67PfluR64PP) were monitored for 12h, with OD_{600} and msfGFP measurements every 15 minutes. Markers and error bars represent the mean value and standard error of four biological replicates every 30 minutes. (**c**,**d**) T7-like phage RNAPs generate high msfGFP expression levels from their putative phage promoter in P. putida KT2440 (2) and P. aeruginosa PAO1 (3). P. putida and P. aeruginosa strains pXRX (negative control), pA0RA0 (T7), pB0RB0 (phi15), pC0RC0 (PPPL-1), pD0RD0 (Pf-10) and pE0RE0 (67PfluR64PP) were induced with 0.3 mM 3mBz, after which the OD_{600} and fluorescent intensity was measured every 15 minutes for 12 hours. The fluorescent intensity was normalized for OD and expressed as equivalent 5(6)-FAM concentration (nM). Markers and error bars represent the mean value and standard error of four biological replicates every 30 minutes.

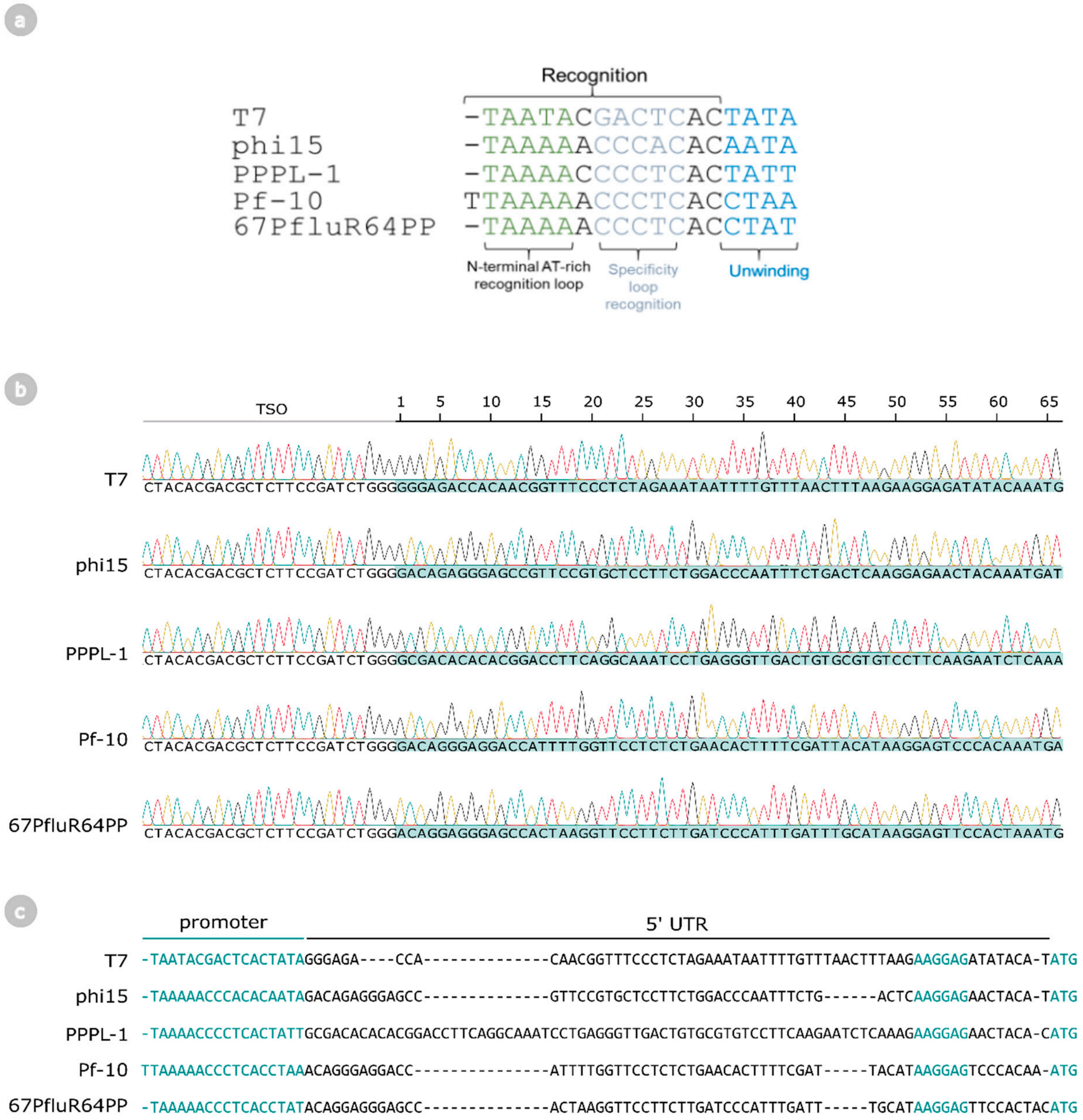

Figure S1. a) Distinct promoter regions of T7-like promoters. The T7 promoter consist of three main regions, 1) an N-terminal AT-rich recognition loop, 2) a specificity loop and 3) an unwinding region. b) Transcription start site (TSS) determination of phage terminators with 5'-capping RACE. The template switching oligonucleotide (TSO) is directly linked to the 5′ terminus of the mRNA transcript and indicates the TSS of the phage promoter. c) Clustal-omega alignment of the validated promoter and 5′ UTR of the phages′ major capsid protein (MCP 5′UTR).

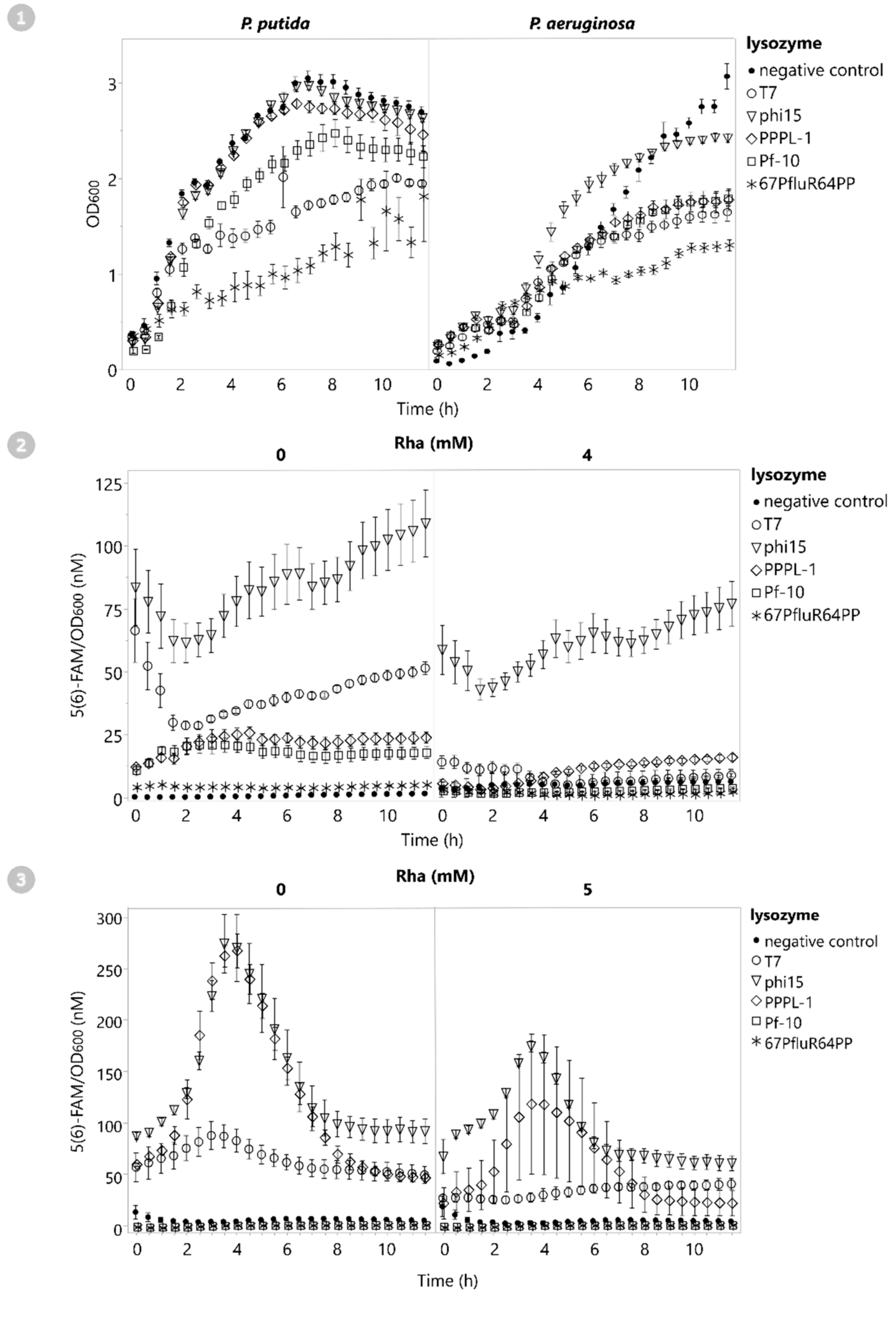

Figure S2. 1) Toxicity assay of phage lysozymes in P. putida KT2440 and P. aeruginosa PAO1. P. putida and P. aeruginosa strains LXO (negative control), LA0 (T7), LB0 (phi15), LC0 (PPPL-1), LD0 (Pf-10) and LE0 (67PfluR64PP) were induced with 10 mM Rha at OD_{600} 0.3, after which cell growth was monitored every half hour for 12h. Datapoints represent the mean OD_{600} value of four biological replicates. Error bars represent the standard error. 2) Fluorescence assay to analyze the inhibitory effect of the phage lysozyme on its corresponding phage RNAP. P. putida strains pXRXLX (negative control), pA0RA0LA0 (T7), pB0RB0LB0 (phi15), pC0RC0LC0 (PPPL-1), pD0RD0LD0 (Pf-10) and pE0RE0LE0 (67PfluR64PP) were induced with 4 mM Rha, after which the fluorescence intensity and cell growth is monitored every half hour for 12h. Datapoints represent the mean 5(6)-FAM/OD_{600} value of four biological replicates. Error bars represent the standard error. 3) Fluorescence assay to analyze

the inhibitory effect of the phage lysozyme on its corresponding phage RNAP. P. aeruginosa strains pXRXLX (negative control), pA0RA0LA0 (T7), pB0RB0LB0 (phi15), pC0RC0LC0 (PPPL-1), pD0RD0LD0 (Pf-10) and pE0RE0LE0 (67PfluR64PP) were induced with 5 mM Rha, after which the fluorescence intensity and cell growth is monitored every half hour for 12h. Datapoints represent the mean 5(6)-FAM/OD_{600} value of four biological replicates. Error bars represent the standard error.

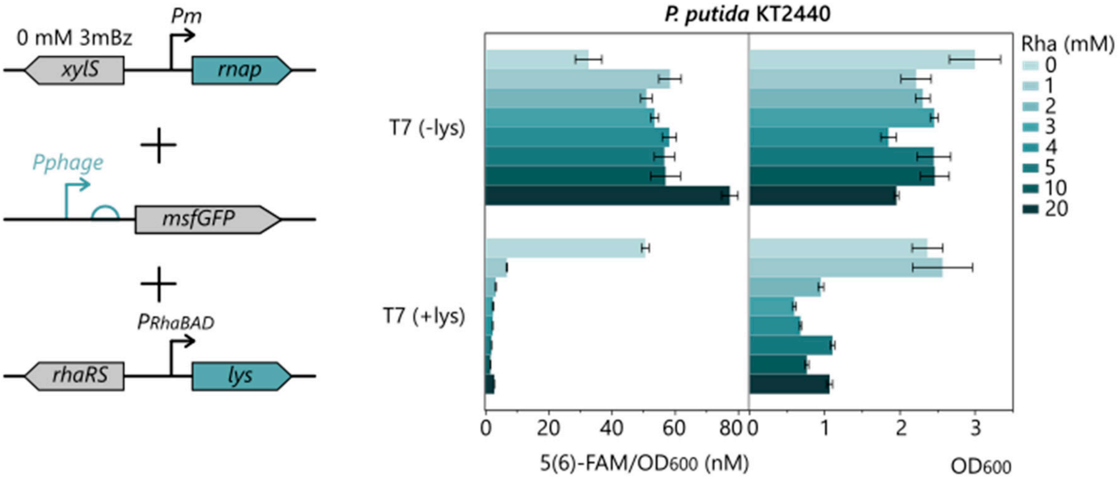

Figure S3. Preliminary assay to determine the ideal rhamnose concentration for induction of phage lysozymes. P. putida strains pA0RA0 (T7(-lys)) and pA0RA0LA0 (T7(+lys)) were induced with 0-20 mM Rha for 12h. Bars and error bars represent the mean 5(6)-FAM/OD_{600} value and standard error of three technical replicates.

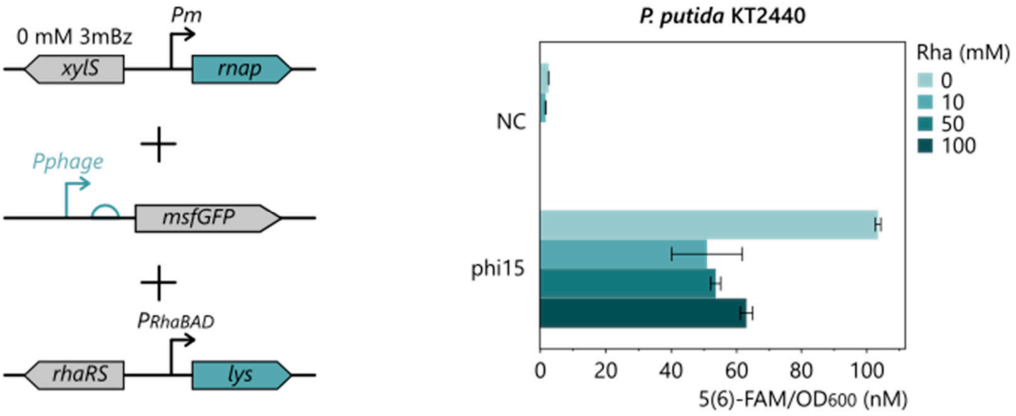

Figure S4. Fluorescence assay to analyze the inhibitory effect of the phi15 lysozyme on its corresponding phage RNAP under different inducer concentrations. P. putida strains pXRXLX (negative control) and pB0RB0LB0 (phi15) were induced with 0-100 mM Rha for 12h. Bars and error bars represent the mean 5(6)-FAM/OD_{600} value and standard error of four biological replicates.

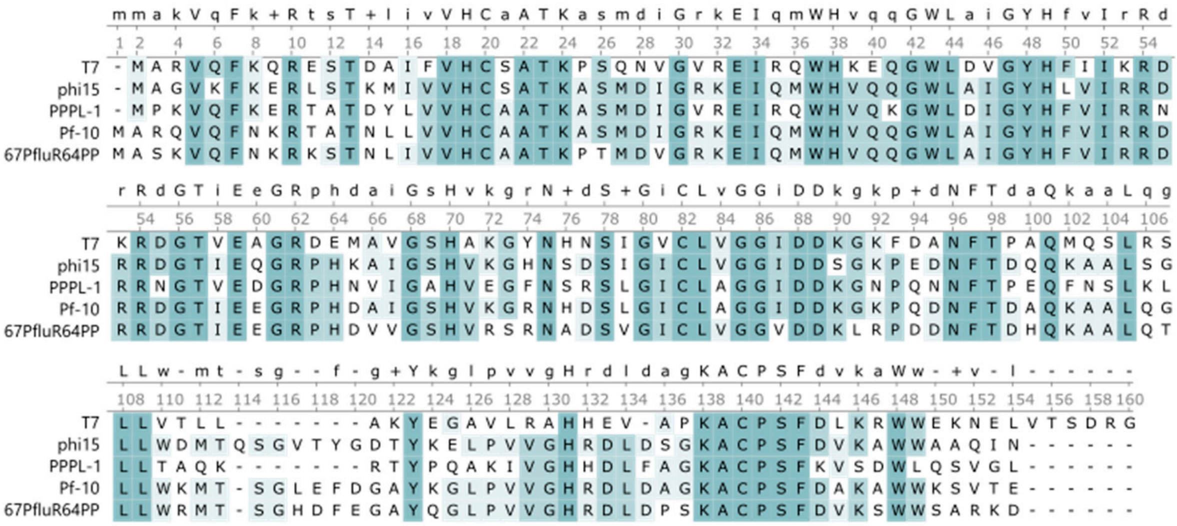

Figure S5. ClustalOmega alignment of phage lysozyme AA sequences. Darker colors indicate higher levels of conservation.

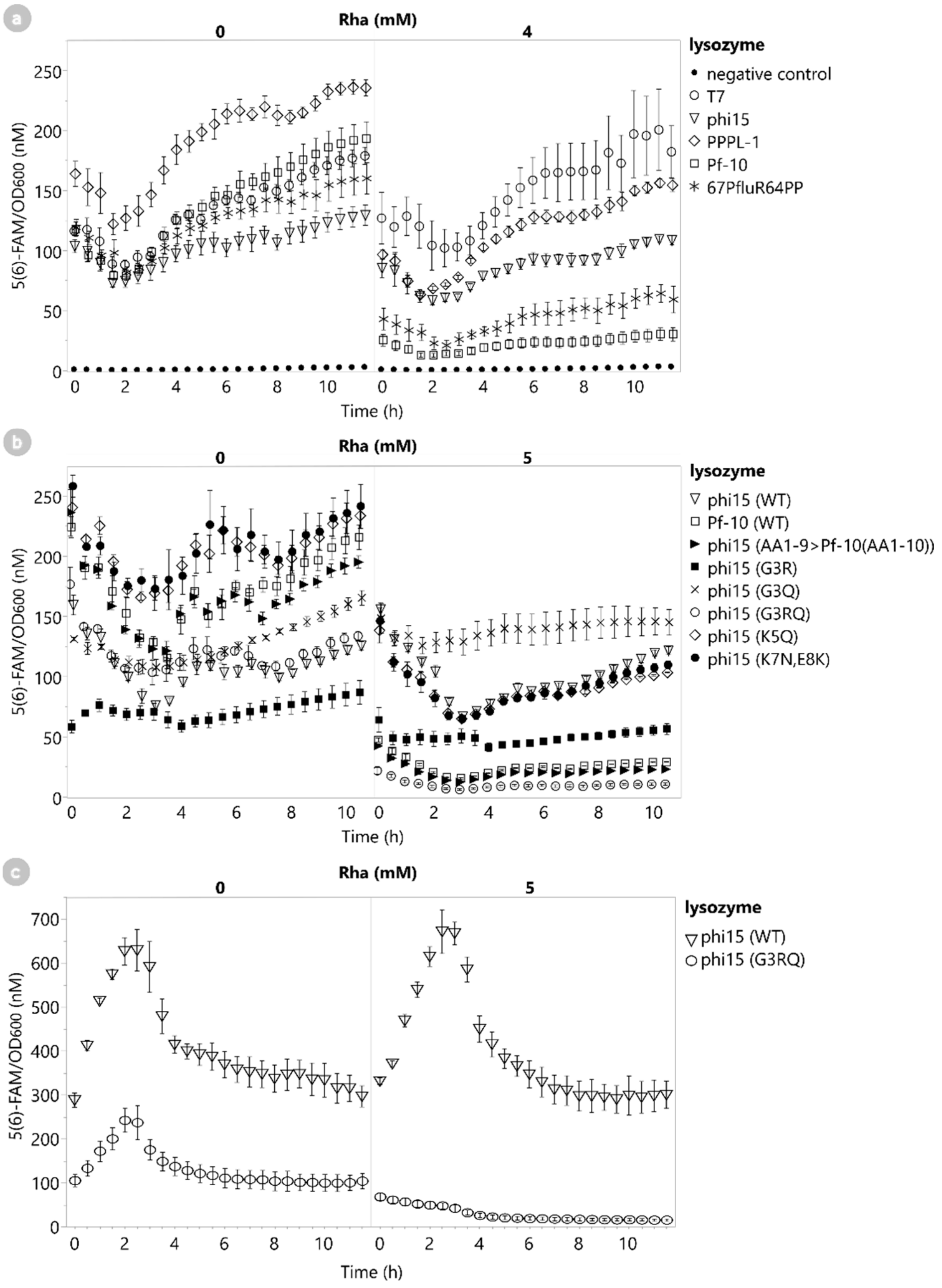

Figure S6. a) Fluorescence intensity assay to analyze the inhibitory effect of different phage lysozyme on phi15 RNAP. P. putida strains pXRXLX (negative control), pB0RB0LA0 (T7), pB0RB0LB0 (phi15), pB0RB0LC0 (PPPL-1), pB0RB0LD0 (Pf-10) and pB0RB0LE0 (67PfluR64PP) were induced with 4 mM Rha at OD_{600} 0.3, after which the fluorescence intensity and cell growth is monitored every half hour for 12h. Datapoints represent the mean 5(6)-FAM/OD_{600} value of four biological replicates. Error bars represent the standard error. b) Fluorescence assay to assess the inhibitory strength of different phi15 lysozyme mutants on phi15 RNAP. P. putida strains pB0RB0LB0 (phi15(WT)), pB0RB0LD0 (Pf-10(WT)), pB0RB0LB1 (phi15(AA1-9>Pf10(AA1-10)), pB0RB0LB2 (phi15(G3R)), pB0RB0LB3 (phi15(G3Q)), pB0RB0LB4 (phi15(G3RQ)), pB0RB0LB5 (phi15(K5Q)) and pB0RB0LB6 (phi15(K7N,E8K)) were induced with 5 mM Rha at OD_{600} 0.3, after which the fluorescence intensity and cell

growth is monitored every half hour for 12h. Datapoints represent the mean 5(6)-FAM/OD_{600} value of four biological replicates. Error bars represent the standard error. c) Fluorescence assay to assess the inhibitory strength of phi15 lysozyme mutant G3RQ on phi15 RNAP. P. aeruginosa strains pB0RB0LB0 (phi15(WT)) and pB0RB0LB4 (phi15(G3RQ)) were induced with 5 mM Rha at OD_{600} 0.3, after which the fluorescence intensity and cell growth is monitored every half hour for 12h. Datapoints represent the mean 5(6)-FAM/OD_{600} value of four biological replicates. Error bars represent the standard error.

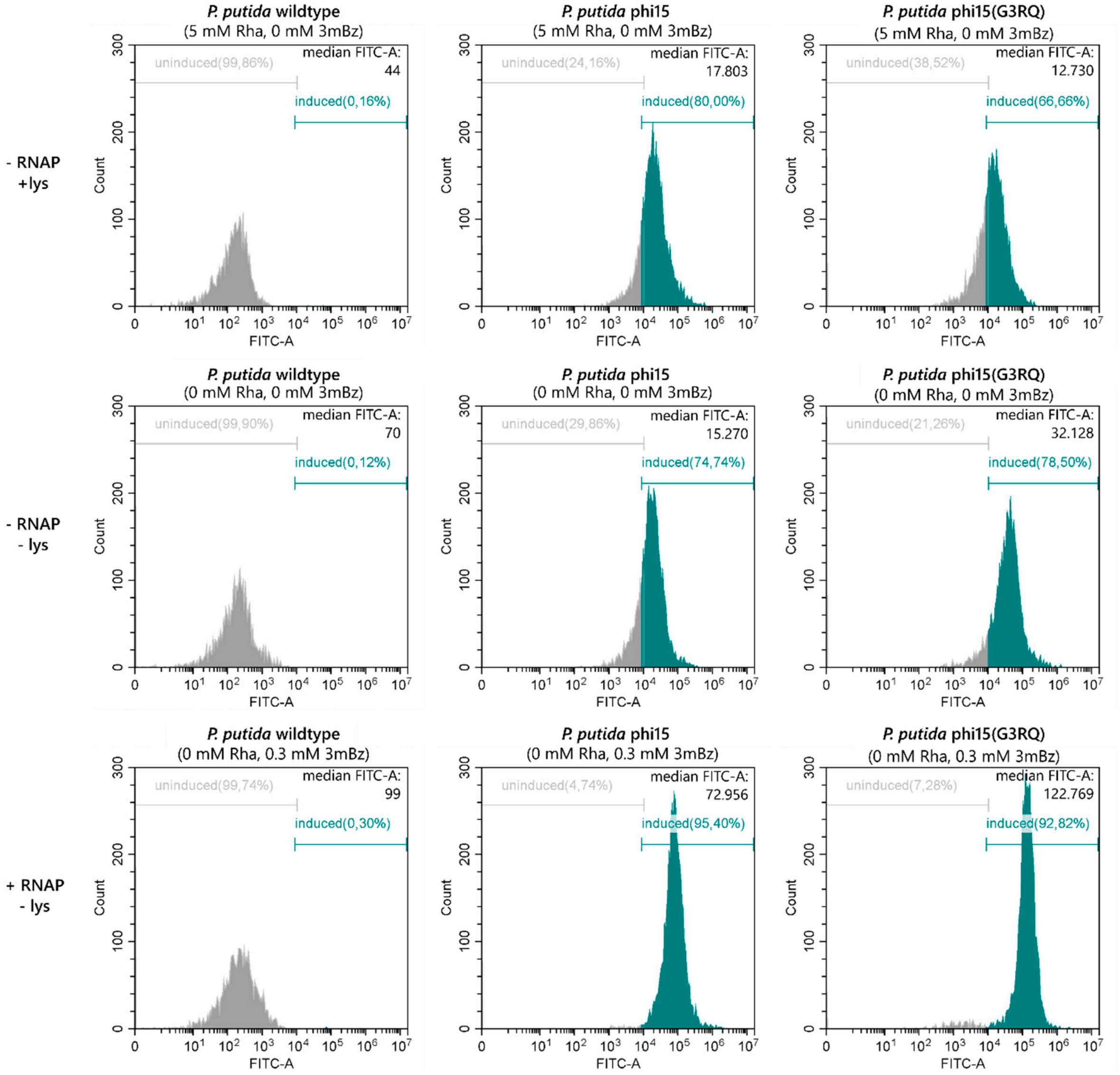

Figure S7. Flow cytometry data of strains P. putida KT2440 (wildtype), P. putida KT2440 with the phi15 RNAP, phi15 reporter construct and phi15 lysozyme (phi15) and P. putida KT2440 with the phi15 RNAP, phi15 reporter construct and phi15 lysozyme (G3RQ) mutant (phi15(G3RQ)). Strains were induced overnight with 5 mM Rha (+lys) or 0.3 mM 3mBz (+RNAP), after which 5,000 cells were analyzed with flow cytometry for FITC-A as described in the method section. Cells with a FITC-A level

above 10^4 are considered induced (green), whereas cells below 10^4 are uninduced. The median FITC-A depicts the median FITC-A value of the entire cell population.

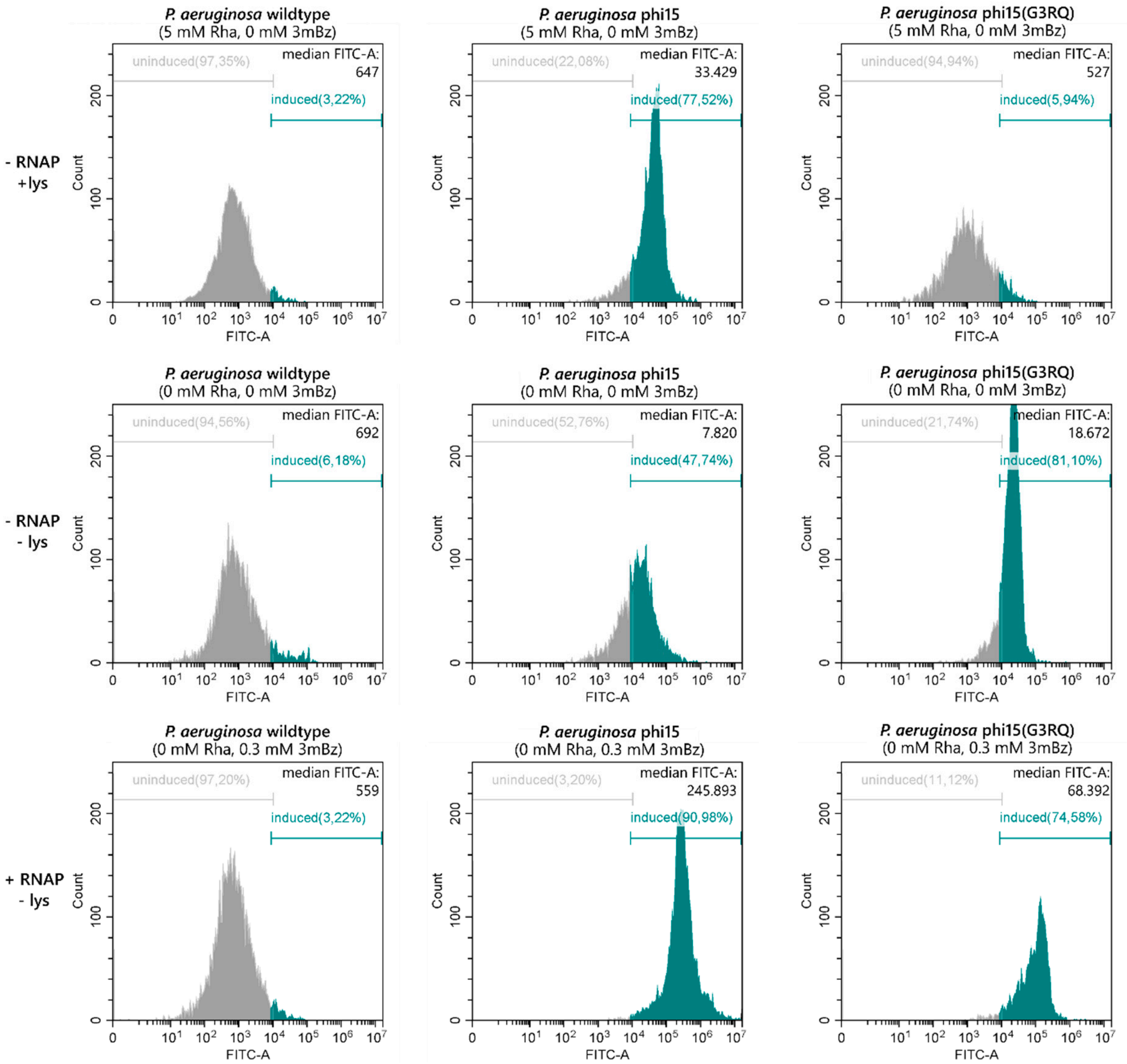

Figure S8. Flow cytometry data of strains P. aeruginosa PAO1 (wildtype), P. aeruginosa PAO1 with the phi15 RNAP, phi15 reporter construct and phi15 lysozyme (phi15) and P. aeruginosa PAO1 with the phi15 RNAP, phi15 reporter construct and phi15 lysozyme (G3RQ) mutant (phi15(G3RQ)). Strains were induced overnight with 5 mM Rha (+lys) or 0.3 mM 3mBz (+RNAP), after which 5,000 cells were analyzed with flow cytometry for FITC-A as described in the method section. Cells with a FITC-A level above 10^4 are considered induced (green), whereas cells below 10^4 are uninduced. The median FITC-A depicts the median FITC-A value of the entire cell population. .

Supplementary Tables

Table S1. Table of primers. Colors of color-coded primers indicate the following. Blue: SapI recognition site; green: SapI restriction site; yellow: BsaI recognition site; grey: BsaI restriction site; red: sequence for amplification of the target region.

Name	Sequence (5′->3′)	Use
SEVA_PS1	AGGGCGGCGGATTTGTCC	Colony PCR and Sanger sequencing of pSTEntry
SEVA_PS2	GCGGCAACCGAGCGTTC	Colony PCR and Sanger sequencing of pSTEntry, pBGDes, pSTDesX and pSTDesR
InsertDes1_F	AATCTGGCTCCCCAACTAATGC	Colony PCR and Sanger sequencing of pSTDesX
InsertDes2_F	GCGTTCGGTCAAGGTTCTGGAC	Colony PCR and Sanger sequencing of pBGDes
InsertDes4_F	CGGCGAAATAGTAATCACGAGGTCAG	Colony PCR and Sanger sequencing of pSTDesR
ST_phi15RNAP_F	CATGACCATGAGGAGGAAAAACAAATGATTGAAGTAGCAAAGAACG	Gibson Assembly of
ST_phi15RNAP_R	CTATCAACAGGAGTCCAAGACTAGTTGTTAGCGAAAGCGAATTGAGAC	pSTDesXa/b·phi15RNAP
ST_Pf10RNAP_F	CATGACCATGAGGAGGAAAAACAAATGGCTGTAATCGAAAAGAAAC	Gibson Assembly of pSTDesXa/b·Pf-10RNAP
ST_Pf10RNAP_R	CTATCAACAGGAGTCCAAGACTAGTTGTTAAGCGAATGCAAACTCGGAC	
ST_PPPL1RNAP_F	CATGACCATGAGGAGGAAAAACAAATGATCCATGAAACTCCGCG	Gibson Assembly of pSTDesXa/b·PPPL-1RNAP
ST_PPPL1RNAP_R	CTATCAACAGGAGTCCAAGACTAGTTGTTATGCGAAAGCGAAGTCAGATTTG	
ST_67PfluR64PPRNAP_F	CATGACCATGAGGAGGAAAAACAAATGGCTATCATCGCACCAG	Gibson Assembly of pSTDesXa/b·67PfluR64PPRNAP
ST_67PfluR64PPPflu_R	CTATCAACAGGAGTCCAAGACTAGTTGTTATGCAAATGCGAATTCAGACTTCAG	
ST_msfGFP_F	GACGCTCTTCCAGAGGTCTCGAATGATCATGGGAATTCATAAAGGTG	Creation of msfGFP
ST_msfGFPend_R	TAGGCTCTTCTCTTGGTCTCATCTTATTTGTAGAGTTCATCCATGCCG	SEVAtile
pBG_BsaI_F	GACGGTCTCTAAGAGAATTCGAGCTCGGTAC	Vector amplification of pBG13 for SEVAtile shuffling
pBG_BsaI_R	GTCGGTCTCCTAGATTAATTAAGACGTCTTGAC	
promRBS_T7_F	GACGCTCTTCCAGAGGTCTCGTCTATAATACGACTCACTATAGG	Creation of $P_{T7,MCP}$
promRBS_T7_R	TAGGCTCTTCTCTTGGTCTCACATTTGTATATCTCCTTC	SEVAtile
promRBS_Phi15_F	GACGCTCTTCCAGAGGTCTCGTCTATAAAAACCCACACAATAG	Creation of $P_{phi15,MCP}$
promRBS_Phi15_R	TAGGCTCTTCTCTTGGTCTCACATTTGTAGTTCTCCTTGAG	SEVAtile
promRBS_PPPL_F	GACGCTCTTCCAGAGGTCTCGTCTATAAAACCCCTCACTATTG	Creation of $P_{PPPL-1,MCP}$
promRBS_PPPL_R	TAGGCTCTTCTCTTGGTCTCACATTTGTAGTTCTCCTTCTTTG	SEVAtile
promRBS_Pf-10_F	GACGCTCTTCCAGAGGTCTCGTCTATTAAAAACCCTCACCTAAAC	Creation of $P_{Pf-10,MCP}$
promRBS_Pf-10_R	TAGGCTCTTCTCTTGGTCTCACATTTGTGGGACTCCTTATGTAATC	SEVAtile
promRBS_67PfluR64PP_F	GACGCTCTTCCAGAGGTCTCGTCTAAAAACCCTCACCTATACAGG	Creation of $P_{67PfluR64PP,MCP}$ SEVAtile
promRBS_67PfluR64PP_R	TAGGCTCTTCTCTTGGTCTCACATTTAGTGGAACTCCTTATGC	
promRBS_T7_F	GACGCTCTTCCAGAGGTCTCGTCTATAATACGACTCACTATAGG	Creation of $P_{T7,GG}$
promRBS_T7_R	TAGGCTCTTCTCTTGGTCTCACATTTGTATATCTCCTTC	SEVAtile
ST_phi15_promb_F	AGAGGTCTCTTCTATAAAAACCCACACAATAGGGCAGTGAGACC	Creation of $P_{phi15,GG}$
ST_phi15_promb_R	CTTGGTCTCACTGCCCTATTGTGTGGGTTTTTATAGAAGAGACC	SEVAtile
ST_PPPL1_promb_F	AGAGGTCTCTTCTATAAAACCCCTCACTATTGGGCAGAGAGACC	Creation of $P_{PPPL-1,GG}$
ST_PPPL1_promb_R	CTTGGTCTCTCTGCCCAATAGTGAGGGGTTTTATAGAAGAGACC	SEVAtile
ST_Pf10_promb_F	AGAGGTCTCTTCTATTAAAAACCCTCACCTAAGGGCAGAGAGACC	Creation of $P_{Pf-10,GG}$
ST_Pf10_promb_R	CTTGGTCTCTCTGCCCTTAGGTGAGGGTTTTTAATAGAAGAGACC	SEVAtile
ST_67PfluR64PP_promb_F	AGAGGTCTCTTCTATAAAAACCCTCACCTATGGGCAGTGAGACC	Creation of $P_{67PfluR64PP,GG}$ SEVAtile
ST_67PfluR64PP_promb_R	CTTGGTCTCACTGCCCATAGGTGAGGGTTTTTATAGAAGAGACC	
ST_phi15_promc_F	AGAGGTCTCTTCTATAAAAACCCACACAATAGAGCAGTGAGACC	Creation of $P_{phi15,GA}$
ST_phi15_promc_R	CTTGGTCTCACTGCTCTATTGTGTGGGTTTTTATAGAAGAGACC	SEVAtile
ST_PPPL1_promc_F	AGAGGTCTCTTCTATAAAACCCCTCACTATTGCGCAGAGAGACC	

Name	Sequence (5′->3′)	Use
ST_PPPL1_promc_R	CTTGGTCTCTCTGCGCAATAGTGAGGGGTTTATAGAAGAGACC	Creation of $P_{\mathrm{PPPL-1,GC}}$ SEVAtile
ST_Pf10_promc_F	AGAGGTCTCTTCTATTAAAAACCCTCACCTAAACGCAGAGAGACC	Creation of $P_{\mathrm{Pf-10,AC}}$ SEVAtile
ST_Pf10_promc_R	CTTGGTCTCTCTGCGTTTAGGTGAGGGTTTTTAATAGAAGAGACC	
ST_67PfluR64PP_promc_F	AGAGGTCTCTTCTATAAAAACCCTCACCTATACGCAGTGAGACC	Creation of $P_{\mathrm{67PfluR64PP,AC}}$ SEVAtile
ST_67PfluR64PP_promc_R	CTTGGTCTCACTGCGTATAGGTGAGGGTTTTTATAGAAGAGACC	
ST_T7_promD_F	AGAGGTCTCTTCTATAATACGACTCACTATAGGGAGACCACAACGCAGAGAGACC	Creation of $P_{\mathrm{T7,MCP(nt1-13)}}$ SEVAtile
ST_T7_promD_R	CTTGGTCTCTCTGCGTTGTGGTCTCCCTATAGTGAGTCGTATTATAGAAGAGACC	
ST_phi15_promD_F	AGAGGTCTCTTCTATAAAAACCCACACAATAGACAGAGGGAGCCGCAGTGAGACC	Creation of $P_{\mathrm{phi15,\ MCP(nt1-13)}}$ SEVAtile
ST_phi15_promD_R	CTTGGTCTCACTGCGGCTCCCTCTGTCTATTGTGTGGGTTTTTATAGAAGAGACC	
ST_PPPL1_promD_F	AGAGGTCTCTTCTATAAAACCCCTCACTATTGCGACACACACGGGCAGAGAGACC	Creation of $P_{\mathrm{PPPL-1,\ MCP(nt1-13)}}$ SEVAtile
ST_PPPL1_promD_R	CTTGGTCTCTCTGCCCGTGTGTGTCGCAATAGTGAGGGGTTTATAGAAGAGACC	
ST_Pf10_promD_F	AGAGGTCTCTTCTATTAAAAACCCTCACCTAAACAGGGAGGACCAGCAGAGAGACC	Creation of $P_{\mathrm{Pf-10,\ MCP(nt1-13)}}$ SEVAtile
ST_Pf10_promD_R	CTTGGTCTCTCTGCTGGTCCTCCCTGTTTAGGTGAGGGTTTTTAATAGAAGAGACC	
ST_67PfluR64PP_promD_F	AGAGGTCTCTTCTATAAAAACCCTCACCTATACAGGAGGGAGCCGCAGTGAGACC	Creation of $P_{\mathrm{67PfluR64PP,\ MCP(nt1-13)}}$ SEVAtile
ST_67PfluR64PP_promD_R	CTTGGTCTCACTGCGGCTCCCTCCTGTATAGGTGAGGGTTTTTATAGAAGAGACC	
ST_BCD2_F	GGCGCTCTTCCAGAGGTCTCTGCAGGCCCAAGTTCACTTAAAAAGG	Creation of BCD2 SEVAtile
ST_BCD2_R3	GCCGCTCTTCTCTTGGTCTCGCATTAGAAAACCTCCTTAGCATG	
ST_T7lys_F	GACGCTCTTCCAGAGGTCTCGAATGGCTCGTGTACAGTTTAAAC	Creation of T7 lysozyme SEVAtile
ST_T7lys_R	TAGGCTCTTCTCTTGGTCTCAGTTATCCACGGTCAGAAGTGACC	
ST_phi15lys_F	GACGCTCTTCCAGAGGTCTCGAATGGCTGGAGTCAAATTTAAGG	Creation of phi15 lysozyme SEVAtile
ST_phi15lys_R	TAGGCTCTTCTCTTGGTCTCAGTTAATTGATCTGAGCGGCCC	
ST_PPPL-1lys_F	GACTGGTCGTAATGAAATTCAGGAGGTGGTCGACAATGCCCAAGGTTCAATTCAAG	Gibson Assembly of pSTDesR·PPPL-1lys
ST_PPPL-1lys_R	CAACAGGAGTCCAAGACTAGGTTACAGGCCGACTGATTGGAGCC	
ST_Pf-10lys_F	GACTGGTCGTAATGAAATTCAGGAGGTGGTCGACAATGGCCCGTCAAGTTCAGTTC	Gibson Assembly of pSTDesR·Pf-10lys
ST_Pf-10lys_R	CAACAGGAGTCCAAGACTAGGTTACTCGGTGACAGACTTCC	
ST_67PfluR64PPlys_F	GACGCTCTTCCAGAGGTCTCGAATGGCGTCCAAAGTGCAGTTC	Creation of 67PfluR64PP lysozyme SEVAtile
ST_67PfluR64PPlys_R	TAGGCTCTTCTCTTGGTCTCAGTTAATCCTTGCGGGCACTCC	
phi15lysAA1-10_F	AGAGGTCTCGAATGGCCCGTCAAGTTCAGTTCAACAAGCGTTTGAGTACCAAGATGATCG	Creation of SEVAtiles of phi15 lysozyme mutants, with reverse primer SNF_ST_phi15lysR
phi15lysG3R_F	TAAGCTCTTCTAGAGGTCTCGAATGGCTCGTGTCAAATTTAAGGAGCGC	
phi15lysG3Q_F	TAAGCTCTTCTAGAGGTCTCGAATGGCTCAAGTCAAATTTAAGGAGCGC	
phi15lysG3RQ_F	AGAGGTCTCGAATGGCTCGTCAAGTCAAATTTAAGGAGCGC	
phi15lysK5Q_F	AGAGGTCTCGAATGGCTGGAGTCCAATTTAAGGAGCGC	
phi15lysKE78NK_F	AGAGGTCTCGAATGGCTGGAGTCAAATTTAACAAGCGCTTGAGTACCAAG	

Table S1: Table of vectors

Name	Relevant features	Reference
pSTDesXa	Destination vector; *oriT*; *oriV(pRO1600/ColE1)*; *xylS/Pm*→ PT3-*SacB*-PT4; Km^R	(Lammens *et al.*, 2021)
pSTDesXa·empty	pSTDesXa in which the *SacB* cassette is substituted with PT3-*linker*-PT4	This work
pSTDesXa·T7RNAP	pSTDesXa in which the *SacB* cassette is substituted with PT3-*T7rnap*-PT4	(Lammens *et al.*, 2021)
pSTDesXa·phi15RNAP	pSTDesXa in which the *SacB* cassette is substituted with PT3-*phi15rnap*-PT4	This work
pSTDesXa·Pf-10RNAP	pSTDesXa in which the *SacB* cassette is substituted with PT3-*Pf-10rnap*-PT4	This work
pSTDesXa·PPPL-1RNAP	pSTDesXa in which the *SacB* cassette is substituted with PT3-*PPPL-1rnap*-PT4	This work
pSTDesXa·67PfluR64PPRNAP	pSTDesXa in which the *SacB* cassette is substituted with PT3-*67PfluR64PPrnap*-PT4	This work
pSTDesXb	Destination vector; *oriT*; *oriV(pRO1600/ColE1)*; *xylS/Pm*→ PT3-*SacB*-PT4; Tc^R	(Lammens *et al.*, 2021)
pSTDesXb·empty	pSTDesXb in which the *SacB* cassette is substituted with PT3-*linker*-PT4	This work
pSTDesXb·T7RNAP	pSTDesXb in which the *SacB* cassette is substituted with PT3-*T7rnap*-PT4	(Lammens *et al.*, 2021)
pSTDesXb·phi15RNAP	pSTDesXb in which the *SacB* cassette is substituted with PT3-*phi15rnap*-PT4	This work
pSTDesXb·Pf-10RNAP	pSTDesXb in which the *SacB* cassette is substituted with PT3-*Pf-10rnap*-PT4	This work
pSTDesXb·PPPL-1RNAP	pSTDesXb in which the *SacB* cassette is substituted with PT3-*PPPL-1rnap*-PT4	This work
pSTDesXb·67PfluR64PPRNAP	pSTDesXb in which the *SacB* cassette is substituted with PT3-*67PfluR64PPrnap*-PT4	This work
pBG13	Destination vector; *oriT*; *oriV(R6K)*; P_{EM7}-*BCD2-msfGFP fusion*; *Tn7L* and *Tn7R* extremes; Km^R/Gm^R	(Zobel *et al.*, 2015)
pBGDes·*BCD2*-msfGFP	pBG13 derivative with PT1-*BCD2*-PT3-*msfGFP*-PT4 fusion	(Lammens *et al.*, 2021)
pBGDes·$P_{T7,MCP}$-msfGFP	pBG13 derivative with PT1-$P_{T7,MCP}$-PT3-*msfGFP*-PT4 fusion	(Lammens *et al.*, 2021)
pBGDes·$P_{phi15,MCP}$-msfGFP	pBG13 derivative with PT1-$P_{phi15,MCP}$-PT3-*msfGFP*-PT4 fusion	This work
pBGDes·$P_{PPPL-1,MCP}$-msfGFP	pBG13 derivative with PT1-$P_{PPPL-1,MCP}$-PT3-*msfGFP*-PT4 fusion	This work
pBGDes·$P_{Pf-10,MCP}$-msfGFP	pBG13 derivative with PT1-$P_{Pf-10-MCP}$-PT3-*msfGFP*-PT4 fusion	This work
pBGDes·$P_{67PfluR64PP,MCP}$-msfGFP	pBG13 derivative with PT1-$P_{67PfluR64PP,MCP}$-PT3-*msfGFP*-PT4 fusion	This work
pBGDes·$P_{T7,GG}$-msfGFP	pBG13 derivative with PT1-$P_{T7,GG}$-PT2-*BCD2*-PT3-*msfGFP*-PT4 fusion	This work
pBGDes·$P_{phi15,GG}$-msfGFP	pBG13 derivative with PT1-$P_{phi15,GG}$-PT2-*BCD2*-PT3-*msfGFP*-PT4 fusion	This work
pBGDes·$P_{PPPL-1,GG}$-msfGFP	pBG13 derivative with PT1-$P_{PPPL-1,GG}$-PT2-*BCD2*-PT3-*msfGFP*-PT4 fusion	This work
pBGDes·$P_{Pf-10,GG}$-msfGFP	pBG13 derivative with PT1-$P_{Pf-10-GG}$-PT2-*BCD2*-PT3-*msfGFP*-PT4 fusion	This work
pBGDes·$P_{67PfluR64PP,GG}$-msfGFP	pBG13 derivative with PT1-$P_{67PfluR64PP,GG}$-PT2-*BCD2*-PT3-*msfGFP*-PT4 fusion	This work
pBGDes·$P_{phi15,GA}$-msfGFP	pBG13 derivative with PT1-$P_{phi15,GA}$-PT2-*BCD2*-PT3-*msfGFP*-PT4 fusion	This work
pBGDes·$P_{PPPL-1,GC}$-msfGFP	pBG13 derivative with PT1-$P_{PPPL-1,GC}$-PT2-*BCD2*-PT3-*msfGFP*-PT4 fusion	This work
pBGDes·$P_{Pf-10,AC}$-msfGFP	pBG13 derivative with PT1-$P_{Pf-10-AC}$-PT2-*BCD2*-PT3-*msfGFP*-PT4 fusion	This work
pBGDes·$P_{67PfluR64PP,AC}$-msfGFP	pBG13 derivative with PT1-$P_{67PfluR64PP,AC}$-PT2-*BCD2*-PT3-*msfGFP*-PT4 fusion	This work
pBGDes·$P_{T7,MCP(NT1-13)}$-msfGFP	pBG13 derivative with PT1-$P_{T7,MCP(nt1-13)}$-PT2-*BCD2*-PT3-*msfGFP*-PT4 fusion	This work
pBGDes·$P_{phi15,MCP(NT1-13)}$-msfGFP	pBG13 derivative with PT1-$P_{phi15,MCP(nt1-13)}$-PT2-*BCD2*-PT3-*msfGFP*-PT4 fusion	This work
pBGDes·$P_{PPPL-1,MCP(NT1-13)}$-msfGFP	pBG13 derivative with PT1-$P_{PPPL-1,MCP(nt1-13)}$-PT2-*BCD2*-PT3-*msfGFP*-PT4 fusion	This work
pBGDes·$P_{Pf-10,MCP(NT1-13)}$-msfGFP	pBG13 derivative with PT1-$P_{Pf-10-MCP(nt1-13)}$-PT2-*BCD2*-PT3-*msfGFP*-PT4 fusion	This work

pBGDes·$P_{67PfluR64PP,MCP(NT1-13)}$-msfGFP	pBG13 derivative with PT1-$P_{67PfluR64PP,MCP(nt1-13)}$-PT2-*BCD2*-PT3-*msfGFP*-PT4 fusion	This work
pSTDesR	Destination vector; *oriT*; *oriV(RK2)*; *RhaRS*/p_{RhaBAD}→MCS; Sm^R/Sp^R	(Lammens *et al.*, 2021)
pSTDesR·msfGFP	pSTDesR in which the MCS is substituted with PT3-*msfGFP*-PT4	(Lammens *et al.*, 2021)
pSTDesR·T7lys	pSTDesR in which the MCS is substituted with PT3-*T7lysozyme*-PT4	(Lammens *et al.*, 2021)
pSTDesR·phi15lys	pSTDesR in which the MCS is substituted with PT3-*phi15lysozyme*-PT4	This work
pSTDesR·PPPL-1lys	pSTDesR in which the MCS is substituted with PT3-*PPPL-1lysozyme*-PT4	This work
pSTDesR·Pf-10lys	pSTDesR in which the MCS is substituted with PT3-*Pf-10lysozyme*-PT4	This work
pSTDesR·67PfluR64PPlys	pSTDesR in which the MCS is substituted with PT3-*67PfmuR64PPlysozyme*-PT4	This work
pSTDesR·phi15lys(AA1-9>Pf-10(AA1-10))	pSTDesR in which the MCS is substituted with PT3-*phi15lysozyme(AA1-9>Pf-10(AA1-10))*-PT4	This work
pSTDesR·phi15lys(G3R)	pSTDesR in which the MCS is substituted with PT3-*phi15lysozyme(G3R)*-PT4	This work
pSTDesR·phi15lys(G3Q)	pSTDesR in which the MCS is substituted with PT3-*phi15lysozyme(G3Q)*-PT4	This work
pSTDesR·phi15lys(G3RQ)	pSTDesR in which the MCS is substituted with PT3-*phi15lysozyme(G3RQ)*-PT4	This work
pSTDesR·phi15lys(K5Q)	pSTDesR in which the MCS is substituted with PT3-*phi15lysozyme(K5Q)*-PT4	This work
pSTDesR·phi15lys(K7N,E8K)	pSTDesR in which the MCS is substituted with PT3-*phi15lysozyme(K7N,E8K)*-PT4	This work
pTNS2	Helper plasmid; *oriV(R6K)*; *tnsABCD*; Amp^R	(Choi *et al.*, 2005)

Table S2: Bacterial strains used in this work. [1]Transformant strains were given a code, depending on the introduced vectors and inserts: p: reporter construct on pBGDes, R: RNAP on pSTDesX, L: lysozyme on pSTDesR. Letters A, B, C, D and E refer to phages T7, phi15, PPPL-1, Pf-10 and 67PfluR64PP, respectively. X refers to empty control vectors. Indices indicate the variant number.

**Name[1]**	**Description**
E. coli TOP10	Intermediate host for vector cloning (Invitrogen™); F- *mcrA* Δ(*mrr-hsd*RMS-*mcr*BC) Φ80*lac*ZΔM15 Δ *lac*X74 *rec*A1 *ara*D139 Δ(*araleu*)7697 *gal*U *gal*K *rps*L (StrR) *end*A1 *nup*G
E. coli PIR2	Intermediate host for cloning of vectors with R6K origin (Invitrogen™); F- *Δlac*169 *rpo*S(am) *rob*A1 *cre*C510 *hsd*R514 *end*A *rec*A1 *uidA(ΔMlu*I)*::pir*
P. putida KT2440	Derivative of *P. putida* mt-2 lacking the TOL plasmid (Bagdasarian *et al.*, 1981)
P. putida RX	Km^R, *P. putida* KT2440 with pSTDesXa·empty
P. putida RA0	Km^R, *P. putida* KT2440 with pSTDesXa·T7RNAP
P. putida RB0	Km^R, *P. putida* KT2440 with pSTDesXa·phi15RNAP
P. putida RC0	Km^R, *P. putida* KT2440 with pSTDesXa·PPPL-1RNAP
P. putida RD0	Km^R, *P. putida* KT2440 with pSTDesXa·Pf-10RNAP
P. putida RE0	Km^R, *P. putida* KT2440 with pSTDesXa·67PfluR64PPRNAP
P. putida pX	Gm^R, *P. putida* KT2440 with genomic insertion of pBGDes·*BCD2*-msfGFP
P. putida pA0	Gm^R, *P. putida* KT2440 with genomic insertion of pBGDes·$P_{T7,MCP}$-msfGFP

Name[1]	Description
P. putida pB0	Gm^R, *P. putida* KT2440 with genomic insertion of pBGDes·$P_{phi15,MCP}$-msfGFP
P. putida pC0	Gm^R, *P. putida* KT2440 with genomic insertion of pBGDes·$P_{PPPL-1,MCP}$-msfGFP
P. putida pD0	Gm^R, *P. putida* KT2440 with genomic insertion of pBGDes·P_{Pf-10}-msfGFP
P. putida pE0	Gm^R, *P. putida* KT2440 with genomic insertion of pBGDes·$P_{67PfluR64PP}$-msfGFP
P. putida pXRX	Gm^R, Km^R, *P. putida* KT2440 with genomic insertion of pBGDes·*BCD2*-msfGFP and pSTDesXa·empty
P. putida pA0RA0	Gm^R, Km^R, *P. putida* KT2440 with genomic insertion of pBGDes·$P_{T7,MCP}$-msfGFP and pSTDesXa·T7RNAP
P. putida pA0RB0	Gm^R, Km^R, *P. putida* KT2440 with genomic insertion of pBGDes·$P_{T7,MCP}$-msfGFP and pSTDesXa·phi15RNAP
P. putida pA0RC0	Gm^R, Km^R, *P. putida* KT2440 with genomic insertion of pBGDes·$P_{T7,MCP}$-msfGFP and pSTDesXa·PPPL-1RNAP
P. putida pA0RD0	Gm^R, Km^R, *P. putida* KT2440 with genomic insertion of pBGDes·$P_{T7,MCP}$-msfGFP and pSTDesXa·Pf-10RNAP
P. putida pA0RE0	Gm^R, Km^R, *P. putida* KT2440 with genomic insertion of pBGDes·$P_{T7,MCP}$-msfGFP and pSTDesXa·67PfluR64PPRNAP
P. putida pA0RA0	Gm^R, Km^R, *P. putida* KT2440 with genomic insertion of pBGDes·$P_{T7,MCP}$-msfGFP and pSTDesXa·T7RNAP
P. putida pB0RA0	Gm^R, Km^R, *P. putida* KT2440 with genomic insertion of pBGDes·$P_{phi15,MCP}$-msfGFP and pSTDesXa·T7RNAP
P. putida pB0RB0	Gm^R, Km^R, *P. putida* KT2440 with genomic insertion of pBGDes·$P_{phi15,MCP}$-msfGFP and pSTDesXa·phi15RNAP
P. putida pB0RC0	Gm^R, Km^R, *P. putida* KT2440 with genomic insertion of pBGDes·$P_{phi15,MCP}$-msfGFP and pSTDesXa·PPPL-1RNAP
P. putida pB0RD0	Gm^R, Km^R, *P. putida* KT2440 with genomic insertion of pBGDes·$P_{phi15,MCP}$-msfGFP and pSTDesXa·Pf-10RNAP
P. putida pB0RE0	Gm^R, Km^R, *P. putida* KT2440 with genomic insertion of pBGDes·$P_{phi15,MCP}$-msfGFP and pSTDesXa·67PfluR64PPRNAP
P. putida pC0RA0	Gm^R, Km^R, *P. putida* KT2440 with genomic insertion of pBGDes·$P_{PPPL-1,MCP}$-msfGFP and pSTDesXa·T7RNAP
P. putida pC0RB0	Gm^R, Km^R, *P. putida* KT2440 with genomic insertion of pBGDes·$P_{PPPL-1,MCP}$-msfGFP and pSTDesXa·phi15RNAP
P. putida pC0RC0	Gm^R, Km^R, *P. putida* KT2440 with genomic insertion of pBGDes·$P_{PPPL-1,MCP}$-msfGFP and pSTDesXa·PPPL-1RNAP
P. putida pC0RD0	Gm^R, Km^R, *P. putida* KT2440 with genomic insertion of pBGDes·$P_{PPPL-1,MCP}$-msfGFP and pSTDesXa·Pf-10RNAP
P. putida pC0RE0	Gm^R, Km^R, *P. putida* KT2440 with genomic insertion of pBGDes·$P_{PPPL-1,MCP}$-msfGFP and pSTDesXa·67PfluR64PPRNAP
P. putida pD0RA0	Gm^R, Km^R, *P. putida* KT2440 with genomic insertion of pBGDes·$P_{Pf-10,MCP}$-msfGFP and pSTDesXa·T7RNAP
P. putida pD0RB0	Gm^R, Km^R, *P. putida* KT2440 with genomic insertion of pBGDes·$P_{Pf-10,MCP}$-msfGFP and pSTDesXa·phi15RNAP
P. putida pD0RC0	Gm^R, Km^R, *P. putida* KT2440 with genomic insertion of pBGDes·$P_{Pf-10,MCP}$-msfGFP and pSTDesXa·PPPL-1RNAP
P. putida pD0RD0	Gm^R, Km^R, *P. putida* KT2440 with genomic insertion of pBGDes·$P_{Pf-10,MCP}$-msfGFP and pSTDesXa·Pf-10RNAP
P. putida pD0RE0	Gm^R, Km^R, *P. putida* KT2440 with genomic insertion of pBGDes·$P_{Pf-10,MCP}$-msfGFP and pSTDesXa·67PfluR64PPRNAP
P. putida pE0RA0	Gm^R, Km^R, *P. putida* KT2440 with genomic insertion of pBGDes·$P_{67PfluR64PP,MCP}$-msfGFP and pSTDesXa·T7RNAP
P. putida pE0RB0	Gm^R, Km^R, *P. putida* KT2440 with genomic insertion of pBGDes·$P_{67PfluR64PP,MCP}$-msfGFP and pSTDesXa·phi15RNAP
P. putida pE0RC0	Gm^R, Km^R, *P. putida* KT2440 with genomic insertion of pBGDes·$P_{67PfluR64PP,MCP}$-msfGFP and pSTDesXa·PPPL-1RNAP
P. putida pE0RD0	Gm^R, Km^R, *P. putida* KT2440 with genomic insertion of pBGDes·$P_{67PfluR64PP,MCP}$-msfGFP and pSTDesXa·Pf-10RNAP

Name[1]	Description
P. putida pE0RE0	Gm^R, Km^R, *P. putida* KT2440 with genomic insertion of pBGDes·$P_{67PfluR64PP,MCP}$-msfGFP and pSTDesXa·67PfluR64PPRNAP
P. putida pA1RA0	Gm^R, Km^R, *P. putida* KT2440 with genomic insertion of pBGDes·$P_{T7,GG}$-msfGFP and pSTDesXa·T7RNAP
P. putida pB1RB0	Gm^R, Km^R, *P. putida* KT2440 with genomic insertion of pBGDes·$P_{phi15,GG}$-msfGFP and pSTDesXa·phi15RNAP
P. putida pC1RC0	Gm^R, Km^R, *P. putida* KT2440 with genomic insertion of pBGDes·$P_{PPPL-1,GG}$-msfGFP and pSTDesXa·PPPL-1RNAP
P. putida pD1RD0	Gm^R, Km^R, *P. putida* KT2440 with genomic insertion of pBGDes·$P_{Pf-10,GG}$-msfGFP and pSTDesXa·Pf-10RNAP
P. putida pE1RE0	Gm^R, Km^R, *P. putida* KT2440 with genomic insertion of pBGDes·$P_{67PfluR64PP,GG}$-msfGFP and pSTDesXa·67PfluR64PPRNAP
P. putida pB2RB0	Gm^R, Km^R, *P. putida* KT2440 with genomic insertion of pBGDes·$P_{phi15,GA}$-msfGFP and pSTDesXa·phi15RNAP
P. putida pC2RC0	Gm^R, Km^R, *P. putida* KT2440 with genomic insertion of pBGDes·$P_{PPPL-1,GC}$-msfGFP and pSTDesXa·PPPL-1RNAP
P. putida pD2RD0	Gm^R, Km^R, *P. putida* KT2440 with genomic insertion of pBGDes·$P_{Pf-10,AC}$-msfGFP and pSTDesXa·Pf-10RNAP
P. putida pE2RE0	Gm^R, Km^R, *P. putida* KT2440 with genomic insertion of pBGDes·$P_{67PfluR64PP,AC}$-msfGFP and pSTDesXa·67PfluR64PPRNAP
P. putida pA3RA0	Gm^R, Km^R, *P. putida* KT2440 with genomic insertion of pBGDes·$P_{T7,MCP(nt1-13)}$-msfGFP and pSTDesXa·T7RNAP
P. putida pB3RB0	Gm^R, Km^R, *P. putida* KT2440 with genomic insertion of pBGDes·$P_{phi15,MCP(nt1-13)}$-msfGFP and pSTDesXa·phi15RNAP
P. putida pC3RC0	Gm^R, Km^R, *P. putida* KT2440 with genomic insertion of pBGDes·$P_{PPPL-1,MCP(nt1-13)}$-msfGFP and pSTDesXa·PPPL-1RNAP
P. putida pD3RD0	Gm^R, Km^R, *P. putida* KT2440 with genomic insertion of pBGDes·$P_{Pf-10,MCP(nt1-13)}$-msfGFP and pSTDesXa·Pf-10RNAP
P. putida pE3RE0	Gm^R, Km^R, *P. putida* KT2440 with genomic insertion of pBGDes·$P_{67PfluR64PP,MCP(nt1-13)}$-msfGFP and pSTDesXa·67PfluR64PPRNAP
P. putida pA0RA1	Gm^R, Km^R, *P. putida* KT2440 with genomic insertion of pBGDes·$P_{T7,MCP}$-msfGFP and pSTDesXa·T7RNAP(R632S)
P. putida pB0RB1	Gm^R, Km^R, *P. putida* KT2440 with genomic insertion of pBGDes·$P_{phi15,MCP}$-msfGFP and pSTDesXa·phi15RNAP(R630S)
P. putida LX	Sm^R, *P. putida* KT2440 with pSTDesR·empty
P. putida LA0	Sm^R, *P. putida* KT2440 with pSTDesR·T7lys
P. putida LB0	Sm^R, *P. putida* KT2440 with pSTDesR·phi15lys
P. putida LC0	Sm^R, *P. putida* KT2440 with pSTDesR·PPPL-1lys
P. putida LD0	Sm^R, *P. putida* KT2440 with pSTDesR·Pf-10lys
P. putida LE0	Sm^R, *P. putida* KT2440 with pSTDesR·67PfluR64PPlys
P. putida pXRXLX	Gm^R, Km^R, Sm^R, *P. putida* KT2440 with genomic insertion of pBGDes·*BCD2*-msfGFP, pSTDesXa·empty and pSTDesR·empty
P. putida pA0RA0LA0	Gm^R, Km^R, Sm^R, *P. putida* KT2440 with genomic insertion of pBGDes·$P_{T7,MCP}$-msfGFP, pSTDesXa·T7RNAP and pSTDesR·T7lys
P. putida pB0RB0LB0	Gm^R, Km^R, Sm^R, *P. putida* KT2440 with genomic insertion of pBGDes·$P_{phi15,MCP}$-msfGFP, pSTDesXa·phi15RNAP and pSTDesR·phi15lys
P. putida pC0RC0LC0	Gm^R, Km^R, Sm^R, *P. putida* KT2440 with genomic insertion of pBGDes·$P_{PPPL-1,MCP}$-msfGFP, pSTDesXa·PPPL-1RNAP and pSTDesR·PPPL-1lys
P. putida pD0RD0LD0	Gm^R, Km^R, Sm^R, *P. putida* KT2440 with genomic insertion of pBGDes·$P_{Pf-10,MCP}$-msfGFP, pSTDesXa·Pf-10RNAP and pSTDesR·Pf-10lys
P. putida pE0RE0LE0	Gm^R, Km^R, Sm^R, *P. putida* KT2440 with genomic insertion of pBGDes·$P_{67PfluR64PP,MCP}$-msfGFP, pSTDesXa·67PfluR64PPRNAP and pSTDesR·67PfluR64PPlys
P. putida pB0RB0LA0	Gm^R, Km^R, Sm^R, *P. putida* KT2440 with genomic insertion of pBGDes·$P_{phi15,MCP}$-msfGFP, pSTDesXa·phi15RNAP and pSTDesR·T7lys

Name[1]	Description
P. putida pB0RB0LC0	Gm^R, Km^R, Sm^R, *P. putida* KT2440 with genomic insertion of pBGDes·$P_{phi15,MCP}$-msfGFP, pSTDesXa·phi15RNAP and pSTDesR·PPPL-1lys
P. putida pB0RB0LD0	Gm^R, Km^R, Sm^R, *P. putida* KT2440 with genomic insertion of pBGDes·$P_{phi15,MCP}$-msfGFP, pSTDesXa·phi15RNAP and pSTDesR·Pf-10lys
P. putida pB0RB0LE0	Gm^R, Km^R, Sm^R, *P. putida* KT2440 with genomic insertion of pBGDes·$P_{phi15,MCP}$-msfGFP, pSTDesXa·phi15RNAP and pSTDesR·67PfluR64PPlys
P. putida pB0RB0LB1	Gm^R, Km^R, Sm^R, *P. putida* KT2440 with genomic insertion of pBGDes·$P_{phi15,MCP}$-msfGFP, pSTDesXa·phi15RNAP and pSTDesR·phi15lys(AA1-9>Pf-10(AA1-10))
P. putida pB0RB0LB2	Gm^R, Km^R, Sm^R, *P. putida* KT2440 with genomic insertion of pBGDes·$P_{phi15,MCP}$-msfGFP, pSTDesXa·phi15RNAP and pSTDesR·phi15lys(G3R)
P. putida pB0RB0LB3	Gm^R, Km^R, Sm^R, *P. putida* KT2440 with genomic insertion of pBGDes·$P_{phi15,MCP}$-msfGFP, pSTDesXa·phi15RNAP and pSTDesR·phi15lys(G3Q)
P. putida pB0RB0LB4	Gm^R, Km^R, Sm^R, *P. putida* KT2440 with genomic insertion of pBGDes·$P_{phi15,MCP}$-msfGFP, pSTDesXa·phi15RNAP and pSTDesR·phi15lys(G3RQ)
P. putida pB0RB0LB5	Gm^R, Km^R, Sm^R, *P. putida* KT2440 with genomic insertion of pBGDes·$P_{phi15,MCP}$-msfGFP, pSTDesXa·phi15RNAP and pSTDesR·phi15lys(K5Q)
P. putida pB0RB0LB6	Gm^R, Km^R, Sm^R, *P. putida* KT2440 with genomic insertion of pBGDes·$P_{phi15,MCP}$-msfGFP, pSTDesXa·phi15RNAP and pSTDesR·phi15lys(K7N,E8K)
P. aeruginosa PAO1	Chloramphenicol-resistant mutant of *P. aeruginosa* isolate PAO (Stover *et al*., 2000)
P. aeruginosa RX	Tc^R, *P. aeruginosa* PA01 with pSTDesXb·empty
P. aeruginosa RA0	Tc^R, *P. aeruginosa* PA01 with pSTDesXb·T7RNAP
P. aeruginosa RB0	Tc^R, *P. aeruginosa* PA01 with pSTDesXb·phi15RNAP
P. aeruginosa RC0	Tc^R, *P. aeruginosa* PA01 with pSTDesXb·PPPL-1RNAP
P. aeruginosa RD0	Tc^R, *P. aeruginosa* PA01 with pSTDesXb·Pf-10RNAP
P. aeruginosa RE0	Tc^R, *P. aeruginosa* PA01 with pSTDesXb·67PfluR64PPRNAP
P. aeruginosa pX	Gm^R, *P. aeruginosa* PAO1 with genomic insertion of pBGDes·*BCD2*-msfGFP
P. aeruginosa pA0	Gm^R, *P. aeruginosa* PAO1 with genomic insertion of pBGDes·$P_{T7,MCP}$-msfGFP
P. aeruginosa pB0	Gm^R, *P. aeruginosa* PAO1 with genomic insertion of pBGDes·$P_{phi15,MCP}$-msfGFP
P. aeruginosa pC0	Gm^R, *P. aeruginosa* PAO1 with genomic insertion of pBGDes·$P_{PPPL-1,MCP}$-msfGFP
P. aeruginosa pD0	Gm^R, *P. aeruginosa* PAO1 with genomic insertion of pBGDes·P_{Pf-10}-msfGFP
P. aeruginosa pE0	Gm^R, *P. aeruginosa* PAO1 with genomic insertion of pBGDes·$P_{67PfluR64PP}$-msfGFP
P. aeruginosa pXRX	Gm^R, Tc^R, *P. aeruginosa* PA01 with genomic insertion of pBGDes·*BCD2*-msfGFP and pSTDesXb·empty
P. aeruginosa pA0RA0	Gm^R, Tc^R, *P. aeruginosa* PA01 with genomic insertion of pBGDes·$P_{T7,MCP}$-msfGFP and pSTDesXb·T7RNAP
P. aeruginosa pA0RB0	Gm^R, Tc^R, *P. aeruginosa* PA01 with genomic insertion of pBGDes·$P_{phi15,MCP}$-msfGFP and pSTDesXb·phi15RNAP
P. aeruginosa pA0RC0	Gm^R, Tc^R, *P. aeruginosa* PA01 with genomic insertion of pBGDes·$P_{PPPL-1,MCP}$-msfGFP and pSTDesXb·PPPL-1RNAP
P. aeruginosa pA0RD0	Gm^R, Tc^R, *P. aeruginosa* PA01 with genomic insertion of pBGDes·$P_{Pf-10,MCP}$-msfGFP and pSTDesXb·Pf-10RNAP
P. aeruginosa pA0RE0	Gm^R, Tc^R, *P. aeruginosa* PA01 with genomic insertion of pBGDes·$P_{67PfluR64PP, MCP}$-msfGFP and pSTDesXb·67PfluR64PPRNAP
P. aeruginosa LX	Sm^R, *P. aeruginosa* PAO1 with pSTDesR·empty
P. aeruginosa LA0	Sm^R, *P. aeruginosa* PAO1 with pSTDesR·T7lys

Name[1]	Description
P. aeruginosa LB0	Sm^R, *P. aeruginosa* PAO1 with pSTDesR·phi15lys
P. aeruginosa LC0	Sm^R, *P. aeruginosa* PAO1 with pSTDesR·PPPL-1lys
P. aeruginosa LD0	Sm^R, *P. aeruginosa* PAO1 with pSTDesR·Pf-10lys
P. aeruginosa LE0	Sm^R, *P. aeruginosa* PAO1 with pSTDesR·67PfluR64PPlys
P. aeruginosa pXRXLX	Gm^R, Tc^R, Sm^R, *P. aeruginosa* PAO1 with genomic insertion of pBGDes·*BCD2*-msfGFP, pSTDesXb·empty and pSTDesR·empty
P. aeruginosa pA0RA0LA0	Gm^R, Tc^R, Sm^R, *P. aeruginosa* PAO1 with genomic insertion of pBGDes·$P_{T7,MCP}$-msfGFP, pSTDesXb·T7RNAP and pSTDesR·T7lys
P. aeruginosa pB0RB0LB0	Gm^R, Tc^R, Sm^R, *P. aeruginosa* PAO1 with genomic insertion of pBGDes·$P_{phi15,MCP}$-msfGFP, pSTDesXb·phi15RNAP and pSTDesR·phi15lys
P. aeruginosa pB0RB0LB4	Gm^R, Tc^R, Sm^R, *P. aeruginosa* PAO1 with genomic insertion of pBGDes·$P_{phi15,MCP}$-msfGFP, pSTDesXb·phi15RNAP and pSTDesR·phi15lys(G3RQ)
P. aeruginosa pC0RC0LC0	Gm^R, Tc^R, Sm^R, *P. aeruginosa* PAO1 with genomic insertion of pBGDes·$P_{PPPL-1,MCP}$-msfGFP, pSTDesXb·PPPL-1RNAP and pSTDesR·PPPL-1lys
P. aeruginosa pD0RD0LD0	Gm^R, Tc^R, Sm^R, *P. aeruginosa* PAO1 with genomic insertion of pBGDes·$P_{Pf-10,MCP}$-msfGFP, pSTDesXb·Pf-10RNAP and pSTDesR·Pf-10lys
P. aeruginosa pE0RE0LE0	Gm^R, Tc^R, Sm^R, *P. aeruginosa* PAO1 with genomic insertion of pBGDes·$P_{67PfluR64PP,MCP}$-msfGFP, pSTDesXb·67PfluR64PPRNAP and pSTDesR·67PfluR64PPlys
P. aeruginosa pB0RB0GD0	Gm^R, Tc^R, Sm^R, *P. aeruginosa* PAO1 with genomic insertion of pBGDes·$P_{phi15,MCP}$-msfGFP, pSTDesXb·phi15RNAP and pSTDesR·empty
P. aeruginosa pB0RB0GD1	Gm^R, Tc^R, Sm^R, *P. aeruginosa* PAO1 with genomic insertion of pBGDes·$P_{phi15,MCP}$-msfGFP, pSTDesXb·phi15RNAP and pSTDesR·LUZ24gp9
P. aeruginosa pB0RB0GD2	Gm^R, Tc^R, Sm^R, *P. aeruginosa* PAO1 with genomic insertion of pBGDes·$P_{phi15,MCP}$-msfGFP, pSTDesXb·phi15RNAP and pSTDesR·LUZ19gp28
P. aeruginosa pB0RB0GD3	Gm^R, Tc^R, Sm^R, *P. aeruginosa* PAO1 with genomic insertion of pBGDes·$P_{phi15,MCP}$-msfGFP, pSTDesXb·phi15RNAP and pSTDesR·phi15gp16

Table S3: All pairwise comparisons using Student's t-test for the cross-recognition assay between phage promoters and RNAPs. This data is summarized in a connecting letters report in Table S5. CL: confidence limit

Promoter-RNAP 1	Promoter-RNAP 2	Difference	Std Err Dif	Lower CL	Upper CL	p-Value
T7 T7	phi15 Pf-10	2058,43	72,51	1913,98	2202,88	<,0001
T7 T7	phi15 PPPL-1	2058,40	72,51	1913,95	2202,85	<,0001
T7 T7	67PfluR64PP PPPL-1	2056,65	72,51	1912,20	2201,10	<,0001
T7 T7	PPPL-1 67PfluR64PP	2053,45	72,51	1908,99	2197,90	<,0001
T7 T7	phi15 67PfluR64PP	2053,33	72,51	1908,88	2197,78	<,0001
T7 T7	67PfluR64PP phi15	2052,99	72,51	1908,53	2197,44	<,0001
T7 T7	Pf-10 T7	2052,06	72,51	1907,61	2196,51	<,0001
T7 T7	PPPL-1 Pf-10	2050,17	72,51	1905,72	2194,63	<,0001
T7 T7	phi15 T7	2048,10	72,51	1903,65	2192,55	<,0001
T7 T7	67PfluR64PP Pf-10	2045,24	72,51	1900,79	2189,69	<,0001
T7 T7	PPPL-1 phi15	2044,75	72,51	1900,30	2189,20	<,0001
T7 T7	T7 Pf-10	2039,53	72,51	1895,08	2183,98	<,0001
T7 T7	67PfluR64PP 67PfluR64PP	2038,37	72,51	1893,91	2182,82	<,0001
T7 T7	T7 67PfluR64PP	2037,92	72,51	1893,47	2182,38	<,0001
T7 T7	Pf-10 67PfluR64PP	2034,65	72,51	1890,20	2179,10	<,0001
T7 T7	Pf-10 phi15	2031,17	72,51	1886,71	2175,62	<,0001
T7 T7	PPPL-1 T7	2031,07	72,51	1886,61	2175,52	<,0001
T7 T7	Pf-10 PPPL-1	2024,45	72,51	1880,00	2168,91	<,0001
T7 T7	T7 PPPL-1	2020,54	72,51	1876,08	2164,99	<,0001
T7 T7	67PfluR64PP T7	2018,68	72,51	1874,22	2163,13	<,0001
T7 T7	T7 phi15	1974,81	72,51	1830,36	2119,26	<,0001
T7 T7	Pf-10 Pf-10	1871,48	72,51	1727,03	2015,93	<,0001
T7 T7	PPPL-1 PPPL-1	1814,85	72,51	1670,40	1959,30	<,0001
T7 T7	phi15 phi15	1400,19	72,51	1255,74	1544,64	<,0001
phi15 phi15	phi15 Pf-10	658,24	72,51	513,79	802,70	<,0001
phi15 phi15	phi15 PPPL-1	658,21	72,51	513,76	802,66	<,0001
phi15 phi15	67PfluR64PP PPPL-1	656,46	72,51	512,01	800,91	<,0001

Promoter-RNAP 1	Promoter-RNAP 2	Difference	Std Err Dif	Lower CL	Upper CL	p-Value
phi15 phi15	PPPL-1 67PfluR64PP	653,26	72,51	508,81	797,71	<,0001
phi15 phi15	phi15 67PfluR64PP	653,14	72,51	508,69	797,59	<,0001
phi15 phi15	67PfluR64PP phi15	652,80	72,51	508,35	797,25	<,0001
phi15 phi15	Pf-10 T7	651,87	72,51	507,42	796,32	<,0001
phi15 phi15	PPPL-1 Pf-10	649,99	72,51	505,53	794,44	<,0001
phi15 phi15	phi15 T7	647,92	72,51	503,46	792,37	<,0001
phi15 phi15	67PfluR64PP Pf-10	645,06	72,51	500,60	789,51	<,0001
phi15 phi15	PPPL-1 phi15	644,56	72,51	500,11	789,01	<,0001
phi15 phi15	T7 Pf-10	639,34	72,51	494,89	783,79	<,0001
phi15 phi15	67PfluR64PP 67PfluR64PP	638,18	72,51	493,73	782,63	<,0001
phi15 phi15	T7 67PfluR64PP	637,74	72,51	493,28	782,19	<,0001
phi15 phi15	Pf-10 67PfluR64PP	634,46	72,51	490,01	778,92	<,0001
phi15 phi15	Pf-10 phi15	630,98	72,51	486,53	775,43	<,0001
phi15 phi15	PPPL-1 T7	630,88	72,51	486,43	775,33	<,0001
phi15 phi15	Pf-10 PPPL-1	624,27	72,51	479,81	768,72	<,0001
phi15 phi15	T7 PPPL-1	620,35	72,51	475,90	764,80	<,0001
phi15 phi15	67PfluR64PP T7	618,49	72,51	474,04	762,94	<,0001
phi15 phi15	T7 phi15	574,62	72,51	430,17	719,07	<,0001
phi15 phi15	Pf-10 Pf-10	471,29	72,51	326,84	615,74	<,0001
phi15 phi15	PPPL-1 PPPL-1	414,66	72,51	270,21	559,11	<,0001
PPPL-1 PPPL-1	phi15 Pf-10	243,59	72,51	99,13	388,04	0,0012
PPPL-1 PPPL-1	phi15 PPPL-1	243,55	72,51	99,10	388,00	0,0012
PPPL-1 PPPL-1	67PfluR64PP PPPL-1	241,80	72,51	97,35	386,25	0,0013
PPPL-1 PPPL-1	PPPL-1 67PfluR64PP	238,60	72,51	94,15	383,05	0,0015
PPPL-1 PPPL-1	phi15 67PfluR64PP	238,48	72,51	94,03	382,93	0,0015
PPPL-1 PPPL-1	67PfluR64PP phi15	238,14	72,51	93,69	382,59	0,0016
PPPL-1 PPPL-1	Pf-10 T7	237,21	72,51	92,76	381,67	0,0016
PPPL-1 PPPL-1	PPPL-1 Pf-10	235,33	72,51	90,88	379,78	0,0018
PPPL-1 PPPL-1	phi15 T7	233,26	72,51	88,80	377,71	0,0019
PPPL-1 PPPL-1	67PfluR64PP Pf-10	230,40	72,51	85,94	374,85	0,0022

Promoter-RNAP 1	Promoter-RNAP 2	Difference	Std Err Dif	Lower CL	Upper CL	p-Value
PPPL-1 PPPL-1	PPPL-1 phi15	229,90	72,51	85,45	374,35	0,0022
PPPL-1 PPPL-1	T7 Pf-10	224,68	72,51	80,23	369,13	0,0027
PPPL-1 PPPL-1	67PfluR64PP 67PfluR64PP	223,52	72,51	79,07	367,97	0,0029
PPPL-1 PPPL-1	T7 67PfluR64PP	223,08	72,51	78,62	367,53	0,0029
PPPL-1 PPPL-1	Pf-10 67PfluR64PP	219,80	72,51	75,35	364,26	0,0033
PPPL-1 PPPL-1	Pf-10 phi15	216,32	72,51	71,87	360,77	0,0038
PPPL-1 PPPL-1	PPPL-1 T7	216,22	72,51	71,77	360,67	0,0039
PPPL-1 PPPL-1	Pf-10 PPPL-1	209,61	72,51	65,16	354,06	0,005
PPPL-1 PPPL-1	T7 PPPL-1	205,69	72,51	61,24	350,14	0,0059
PPPL-1 PPPL-1	67PfluR64PP T7	203,83	72,51	59,38	348,28	0,0063
Pf-10 Pf-10	phi15 Pf-10	186,95	72,51	42,50	331,41	0,0119
Pf-10 Pf-10	phi15 PPPL-1	186,92	72,51	42,47	331,37	0,0119
Pf-10 Pf-10	67PfluR64PP PPPL-1	185,17	72,51	40,72	329,62	0,0127
Pf-10 Pf-10	PPPL-1 67PfluR64PP	181,97	72,51	37,52	326,42	0,0142
Pf-10 Pf-10	phi15 67PfluR64PP	181,85	72,51	37,40	326,30	0,0143
Pf-10 Pf-10	67PfluR64PP phi15	181,51	72,51	37,06	325,96	0,0145
Pf-10 Pf-10	Pf-10 T7	180,58	72,51	36,13	325,03	0,015
Pf-10 Pf-10	PPPL-1 Pf-10	178,70	72,51	34,24	323,15	0,016
Pf-10 Pf-10	phi15 T7	176,63	72,51	32,17	321,08	0,0172
Pf-10 Pf-10	67PfluR64PP Pf-10	173,77	72,51	29,31	318,22	0,0191
Pf-10 Pf-10	PPPL-1 phi15	173,27	72,51	28,82	317,72	0,0194
Pf-10 Pf-10	T7 Pf-10	168,05	72,51	23,60	312,50	0,0232
Pf-10 Pf-10	67PfluR64PP 67PfluR64PP	166,89	72,51	22,44	311,34	0,0241
Pf-10 Pf-10	T7 67PfluR64PP	166,45	72,51	21,99	310,90	0,0245
Pf-10 Pf-10	Pf-10 67PfluR64PP	163,17	72,51	18,72	307,63	0,0274
PPPL-1 PPPL-1	T7 phi15	159,96	72,51	15,51	304,41	0,0304
Pf-10 Pf-10	Pf-10 phi15	159,69	72,51	15,24	304,14	0,0307
Pf-10 Pf-10	PPPL-1 T7	159,59	72,51	15,14	304,04	0,0308
Pf-10 Pf-10	Pf-10 PPPL-1	152,98	72,51	8,52	297,43	0,0382
Pf-10 Pf-10	T7 PPPL-1	149,06	72,51	4,61	293,51	0,0433

Promoter-RNAP 1	Promoter-RNAP 2	Difference	Std Err Dif	Lower CL	Upper CL	p-Value
Pf-10 Pf-10	67PfluR64PP T7	147,20	72,51	2,75	291,65	0,0459
Pf-10 Pf-10	T7 phi15	103,33	72,51	-41,12	247,78	0,1583
T7 phi15	phi15 Pf-10	83,62	72,51	-60,83	228,08	0,2525
T7 phi15	phi15 PPPL-1	83,59	72,51	-60,86	228,04	0,2527
T7 phi15	67PfluR64PP PPPL-1	81,84	72,51	-62,61	226,29	0,2626
T7 phi15	PPPL-1 67PfluR64PP	78,64	72,51	-65,81	223,09	0,2816
T7 phi15	phi15 67PfluR64PP	78,52	72,51	-65,93	222,97	0,2824
T7 phi15	67PfluR64PP phi15	78,18	72,51	-66,28	222,63	0,2844
T7 phi15	Pf-10 T7	77,25	72,51	-67,20	221,70	0,2901
T7 phi15	PPPL-1 Pf-10	75,37	72,51	-69,09	219,82	0,302
T7 phi15	phi15 T7	73,29	72,51	-71,16	217,75	0,3154
T7 phi15	67PfluR64PP Pf-10	70,43	72,51	-74,02	214,89	0,3345
T7 phi15	PPPL-1 phi15	69,94	72,51	-74,51	214,39	0,3379
T7 phi15	T7 Pf-10	64,72	72,51	-79,73	209,17	0,375
T7 phi15	67PfluR64PP 67PfluR64PP	63,56	72,51	-80,89	208,01	0,3836
T7 phi15	T7 67PfluR64PP	63,11	72,51	-81,34	207,57	0,3869
T7 phi15	Pf-10 67PfluR64PP	59,84	72,51	-84,61	204,29	0,4118
PPPL-1 PPPL-1	Pf-10 Pf-10	56,63	72,51	-87,82	201,08	0,4373
T7 phi15	Pf-10 phi15	56,36	72,51	-88,10	200,81	0,4395
T7 phi15	PPPL-1 T7	56,26	72,51	-88,19	200,71	0,4403
T7 phi15	Pf-10 PPPL-1	49,65	72,51	-94,81	194,10	0,4957
T7 phi15	T7 PPPL-1	45,73	72,51	-98,73	190,18	0,5302
T7 phi15	67PfluR64PP T7	43,87	72,51	-100,58	188,32	0,547
67PfluR64PP T7	phi15 Pf-10	39,76	72,51	-104,70	184,21	0,5851
67PfluR64PP T7	phi15 PPPL-1	39,72	72,51	-104,73	184,18	0,5854
67PfluR64PP T7	67PfluR64PP PPPL-1	37,97	72,51	-106,48	182,43	0,602
T7 PPPL-1	phi15 Pf-10	37,90	72,51	-106,55	182,35	0,6028
T7 PPPL-1	phi15 PPPL-1	37,87	72,51	-106,59	182,32	0,6031
T7 PPPL-1	67PfluR64PP PPPL-1	36,12	72,51	-108,34	180,57	0,6199
67PfluR64PP T7	PPPL-1 67PfluR64PP	34,77	72,51	-109,68	179,22	0,633

Promoter-RNAP 1	Promoter-RNAP 2	Difference	Std Err Dif	Lower CL	Upper CL	p-Value
67PfluR64PP T7	phi15 67PfluR64PP	34,65	72,51	-109,80	179,10	0,6341
67PfluR64PP T7	67PfluR64PP phi15	34,31	72,51	-110,14	178,76	0,6375
Pf-10 PPPL-1	phi15 Pf-10	33,98	72,51	-110,47	178,43	0,6407
Pf-10 PPPL-1	phi15 PPPL-1	33,95	72,51	-110,51	178,40	0,641
67PfluR64PP T7	Pf-10 T7	33,39	72,51	-111,07	177,84	0,6466
T7 PPPL-1	PPPL-1 67PfluR64PP	32,91	72,51	-111,54	177,36	0,6512
T7 PPPL-1	phi15 67PfluR64PP	32,79	72,51	-111,66	177,24	0,6524
T7 PPPL-1	67PfluR64PP phi15	32,45	72,51	-112,00	176,90	0,6558
Pf-10 PPPL-1	67PfluR64PP PPPL-1	32,20	72,51	-112,26	176,65	0,6583
T7 PPPL-1	Pf-10 T7	31,53	72,51	-112,93	175,98	0,665
67PfluR64PP T7	PPPL-1 Pf-10	31,50	72,51	-112,95	175,95	0,6653
T7 PPPL-1	PPPL-1 Pf-10	29,64	72,51	-114,81	174,09	0,6839
67PfluR64PP T7	phi15 T7	29,43	72,51	-115,02	173,88	0,686
Pf-10 PPPL-1	PPPL-1 67PfluR64PP	28,99	72,51	-115,46	173,44	0,6904
Pf-10 PPPL-1	phi15 67PfluR64PP	28,87	72,51	-115,58	173,33	0,6916
Pf-10 PPPL-1	67PfluR64PP phi15	28,53	72,51	-115,92	172,98	0,6951
Pf-10 PPPL-1	Pf-10 T7	27,61	72,51	-116,84	172,06	0,7045
T7 PPPL-1	phi15 T7	27,57	72,51	-116,88	172,02	0,7049
PPPL-1 T7	phi15 Pf-10	27,37	72,51	-117,09	171,82	0,7069
PPPL-1 T7	phi15 PPPL-1	27,33	72,51	-117,12	171,79	0,7073
Pf-10 phi15	phi15 Pf-10	27,27	72,51	-117,18	171,72	0,708
Pf-10 phi15	phi15 PPPL-1	27,24	72,51	-117,22	171,69	0,7083
67PfluR64PP T7	67PfluR64PP Pf-10	26,57	72,51	-117,89	171,02	0,7151
67PfluR64PP T7	PPPL-1 phi15	26,07	72,51	-118,38	170,53	0,7202
Pf-10 PPPL-1	PPPL-1 Pf-10	25,72	72,51	-118,73	170,17	0,7238
PPPL-1 T7	67PfluR64PP PPPL-1	25,58	72,51	-118,87	170,04	0,7252
Pf-10 phi15	67PfluR64PP PPPL-1	25,49	72,51	-118,97	169,94	0,7262
T7 PPPL-1	67PfluR64PP Pf-10	24,71	72,51	-119,74	169,16	0,7343
T7 PPPL-1	PPPL-1 phi15	24,22	72,51	-120,24	168,67	0,7394
Pf-10 67PfluR64PP	phi15 Pf-10	23,78	72,51	-120,67	168,23	0,7439

Promoter-RNAP 1	Promoter-RNAP 2	Difference	Std Err Dif	Lower CL	Upper CL	p-Value
Pf-10 67PfluR64PP	phi15 PPPL-1	23,75	72,51	-120,70	168,20	0,7442
Pf-10 PPPL-1	phi15 T7	23,65	72,51	-120,80	168,10	0,7452
PPPL-1 T7	PPPL-1 67PfluR64PP	22,38	72,51	-122,07	166,83	0,7585
Pf-10 phi15	PPPL-1 67PfluR64PP	22,28	72,51	-122,17	166,73	0,7595
PPPL-1 T7	phi15 67PfluR64PP	22,26	72,51	-122,19	166,71	0,7597
Pf-10 phi15	phi15 67PfluR64PP	22,16	72,51	-122,29	166,61	0,7607
Pf-10 67PfluR64PP	67PfluR64PP PPPL-1	22,00	72,51	-122,45	166,45	0,7624
PPPL-1 T7	67PfluR64PP phi15	21,92	72,51	-122,53	166,37	0,7633
Pf-10 phi15	67PfluR64PP phi15	21,82	72,51	-122,63	166,27	0,7643
PPPL-1 T7	Pf-10 T7	21,00	72,51	-123,46	165,45	0,773
Pf-10 phi15	Pf-10 T7	20,90	72,51	-123,56	165,35	0,774
67PfluR64PP T7	T7 Pf-10	20,85	72,51	-123,60	165,30	0,7745
Pf-10 PPPL-1	67PfluR64PP Pf-10	20,79	72,51	-123,66	165,24	0,7751
T7 67PfluR64PP	phi15 Pf-10	20,51	72,51	-123,94	164,96	0,7781
T7 67PfluR64PP	phi15 PPPL-1	20,48	72,51	-123,97	164,93	0,7784
Pf-10 PPPL-1	PPPL-1 phi15	20,30	72,51	-124,16	164,75	0,7803
67PfluR64PP 67PfluR64PP	phi15 Pf-10	20,07	72,51	-124,39	164,52	0,7828
67PfluR64PP 67PfluR64PP	phi15 PPPL-1	20,03	72,51	-124,42	164,49	0,7831
67PfluR64PP T7	67PfluR64PP 67PfluR64PP	19,69	72,51	-124,76	164,14	0,7867
67PfluR64PP T7	T7 67PfluR64PP	19,25	72,51	-125,20	163,70	0,7914
PPPL-1 T7	PPPL-1 Pf-10	19,11	72,51	-125,34	163,56	0,7929
Pf-10 phi15	PPPL-1 Pf-10	19,01	72,51	-125,44	163,46	0,7939
T7 PPPL-1	T7 Pf-10	18,99	72,51	-125,46	163,44	0,7941
T7 Pf-10	phi15 Pf-10	18,91	72,51	-125,55	163,36	0,795
T7 Pf-10	phi15 PPPL-1	18,87	72,51	-125,58	163,32	0,7954
Pf-10 67PfluR64PP	PPPL-1 67PfluR64PP	18,79	72,51	-125,66	163,25	0,7962
T7 67PfluR64PP	67PfluR64PP PPPL-1	18,73	72,51	-125,72	163,18	0,7969
Pf-10 67PfluR64PP	phi15 67PfluR64PP	18,68	72,51	-125,78	163,13	0,7975
Pf-10 67PfluR64PP	67PfluR64PP phi15	18,33	72,51	-126,12	162,79	0,8011
67PfluR64PP 67PfluR64PP	67PfluR64PP PPPL-1	18,28	72,51	-126,17	162,74	0,8016

Promoter-RNAP 1	Promoter-RNAP 2	Difference	Std Err Dif	Lower CL	Upper CL	p-Value
T7 PPPL-1	67PfluR64PP 67PfluR64PP	17,83	72,51	-126,62	162,28	0,8064
Pf-10 67PfluR64PP	Pf-10 T7	17,41	72,51	-127,04	161,86	0,8109
T7 PPPL-1	T7 67PfluR64PP	17,39	72,51	-127,06	161,84	0,8111
T7 Pf-10	67PfluR64PP PPPL-1	17,12	72,51	-127,33	161,57	0,814
PPPL-1 T7	phi15 T7	17,04	72,51	-127,41	161,49	0,8149
Pf-10 phi15	phi15 T7	16,94	72,51	-127,51	161,39	0,8159
67PfluR64PP T7	Pf-10 67PfluR64PP	15,98	72,51	-128,48	160,43	0,8262
T7 67PfluR64PP	PPPL-1 67PfluR64PP	15,52	72,51	-128,93	159,97	0,8311
Pf-10 67PfluR64PP	PPPL-1 Pf-10	15,52	72,51	-128,93	159,97	0,8311
T7 67PfluR64PP	phi15 67PfluR64PP	15,40	72,51	-129,05	159,86	0,8323
67PfluR64PP 67PfluR64PP	PPPL-1 67PfluR64PP	15,08	72,51	-129,37	159,53	0,8358
Pf-10 PPPL-1	T7 Pf-10	15,07	72,51	-129,38	159,53	0,8359
T7 67PfluR64PP	67PfluR64PP phi15	15,06	72,51	-129,39	159,51	0,836
67PfluR64PP 67PfluR64PP	phi15 67PfluR64PP	14,96	72,51	-129,49	159,41	0,8371
67PfluR64PP 67PfluR64PP	67PfluR64PP phi15	14,62	72,51	-129,83	159,07	0,8408
PPPL-1 T7	67PfluR64PP Pf-10	14,18	72,51	-130,28	158,63	0,8455
T7 67PfluR64PP	Pf-10 T7	14,14	72,51	-130,31	158,59	0,8459
T7 PPPL-1	Pf-10 67PfluR64PP	14,12	72,51	-130,33	158,57	0,8462
Pf-10 phi15	67PfluR64PP Pf-10	14,08	72,51	-130,37	158,53	0,8466
T7 Pf-10	PPPL-1 67PfluR64PP	13,92	72,51	-130,53	158,37	0,8483
Pf-10 PPPL-1	67PfluR64PP 67PfluR64PP	13,91	72,51	-130,54	158,36	0,8484
T7 Pf-10	phi15 67PfluR64PP	13,80	72,51	-130,65	158,25	0,8496
67PfluR64PP 67PfluR64PP	Pf-10 T7	13,69	72,51	-130,76	158,15	0,8507
PPPL-1 T7	PPPL-1 phi15	13,68	72,51	-130,77	158,14	0,8508
PPPL-1 phi15	phi15 Pf-10	13,68	72,51	-130,77	158,13	0,8508
PPPL-1 phi15	phi15 PPPL-1	13,65	72,51	-130,80	158,10	0,8512
Pf-10 phi15	PPPL-1 phi15	13,59	72,51	-130,87	158,04	0,8519
Pf-10 PPPL-1	T7 67PfluR64PP	13,47	72,51	-130,98	157,92	0,8531
T7 Pf-10	67PfluR64PP phi15	13,46	72,51	-130,99	157,91	0,8533
Pf-10 67PfluR64PP	phi15 T7	13,45	72,51	-131,00	157,90	0,8533

Promoter-RNAP 1	Promoter-RNAP 2	Difference	Std Err Dif	Lower CL	Upper CL	p-Value
67PfluR64PP Pf-10	phi15 Pf-10	13,19	72,51	-131,26	157,64	0,8562
67PfluR64PP Pf-10	phi15 PPPL-1	13,16	72,51	-131,29	157,61	0,8565
T7 Pf-10	Pf-10 T7	12,53	72,51	-131,92	156,99	0,8632
67PfluR64PP T7	Pf-10 phi15	12,49	72,51	-131,96	156,94	0,8637
67PfluR64PP T7	PPPL-1 T7	12,39	72,51	-132,06	156,84	0,8648
T7 67PfluR64PP	PPPL-1 Pf-10	12,25	72,51	-132,20	156,70	0,8663
PPPL-1 phi15	67PfluR64PP PPPL-1	11,90	72,51	-132,55	156,35	0,8701
67PfluR64PP 67PfluR64PP	PPPL-1 Pf-10	11,81	72,51	-132,64	156,26	0,8711
67PfluR64PP Pf-10	67PfluR64PP PPPL-1	11,41	72,51	-133,04	155,86	0,8754
T7 Pf-10	PPPL-1 Pf-10	10,65	72,51	-133,80	155,10	0,8837
T7 PPPL-1	Pf-10 phi15	10,63	72,51	-133,82	155,08	0,8838
Pf-10 67PfluR64PP	67PfluR64PP Pf-10	10,59	72,51	-133,86	155,04	0,8843
T7 PPPL-1	PPPL-1 T7	10,53	72,51	-133,92	154,98	0,8849
phi15 T7	phi15 Pf-10	10,33	72,51	-134,12	154,78	0,8871
phi15 T7	phi15 PPPL-1	10,30	72,51	-134,15	154,75	0,8875
Pf-10 PPPL-1	Pf-10 67PfluR64PP	10,20	72,51	-134,25	154,65	0,8885
T7 67PfluR64PP	phi15 T7	10,18	72,51	-134,27	154,63	0,8887
Pf-10 67PfluR64PP	PPPL-1 phi15	10,10	72,51	-134,35	154,55	0,8896
67PfluR64PP 67PfluR64PP	phi15 T7	9,74	72,51	-134,71	154,19	0,8935
PPPL-1 phi15	PPPL-1 67PfluR64PP	8,70	72,51	-135,76	153,15	0,9049
PPPL-1 phi15	phi15 67PfluR64PP	8,58	72,51	-135,87	153,03	0,9062
T7 Pf-10	phi15 T7	8,58	72,51	-135,88	153,03	0,9062
phi15 T7	67PfluR64PP PPPL-1	8,55	72,51	-135,90	153,00	0,9065
PPPL-1 T7	T7 Pf-10	8,46	72,51	-135,99	152,91	0,9074
Pf-10 phi15	T7 Pf-10	8,36	72,51	-136,09	152,81	0,9085
PPPL-1 Pf-10	phi15 Pf-10	8,26	72,51	-136,19	152,71	0,9096
PPPL-1 phi15	67PfluR64PP phi15	8,24	72,51	-136,22	152,69	0,9099
PPPL-1 Pf-10	phi15 PPPL-1	8,23	72,51	-136,23	152,68	0,91
67PfluR64PP Pf-10	PPPL-1 67PfluR64PP	8,20	72,51	-136,25	152,66	0,9102
67PfluR64PP Pf-10	phi15 67PfluR64PP	8,09	72,51	-136,37	152,54	0,9115

Promoter-RNAP 1	Promoter-RNAP 2	Difference	Std Err Dif	Lower CL	Upper CL	p-Value
67PfluR64PP Pf-10	67PfluR64PP phi15	7,74	72,51	-136,71	152,20	0,9152
T7 67PfluR64PP	67PfluR64PP Pf-10	7,32	72,51	-137,13	151,77	0,9199
PPPL-1 phi15	Pf-10 T7	7,31	72,51	-137,14	151,76	0,92
PPPL-1 T7	67PfluR64PP 67PfluR64PP	7,30	72,51	-137,15	151,75	0,9201
Pf-10 phi15	67PfluR64PP 67PfluR64PP	7,20	72,51	-137,25	151,65	0,9212
67PfluR64PP 67PfluR64PP	67PfluR64PP Pf-10	6,88	72,51	-137,58	151,33	0,9247
PPPL-1 T7	T7 67PfluR64PP	6,86	72,51	-137,59	151,31	0,9249
T7 67PfluR64PP	PPPL-1 phi15	6,83	72,51	-137,62	151,28	0,9252
67PfluR64PP Pf-10	Pf-10 T7	6,82	72,51	-137,63	151,27	0,9253
Pf-10 phi15	T7 67PfluR64PP	6,76	72,51	-137,69	151,21	0,926
Pf-10 PPPL-1	Pf-10 phi15	6,71	72,51	-137,74	151,16	0,9265
Pf-10 PPPL-1	PPPL-1 T7	6,61	72,51	-137,84	151,06	0,9276
PPPL-1 Pf-10	67PfluR64PP PPPL-1	6,48	72,51	-137,98	150,93	0,9291
67PfluR64PP 67PfluR64PP	PPPL-1 phi15	6,38	72,51	-138,07	150,84	0,9301
Pf-10 T7	phi15 Pf-10	6,37	72,51	-138,08	150,82	0,9302
Pf-10 T7	phi15 PPPL-1	6,34	72,51	-138,11	150,79	0,9306
67PfluR64PP T7	Pf-10 PPPL-1	5,78	72,51	-138,67	150,23	0,9367
T7 Pf-10	67PfluR64PP Pf-10	5,72	72,51	-138,74	150,17	0,9374
67PfluR64PP phi15	phi15 Pf-10	5,45	72,51	-139,00	149,90	0,9403
PPPL-1 phi15	PPPL-1 Pf-10	5,42	72,51	-139,03	149,88	0,9406
67PfluR64PP phi15	phi15 PPPL-1	5,41	72,51	-139,04	149,87	0,9407
phi15 T7	PPPL-1 67PfluR64PP	5,34	72,51	-139,11	149,79	0,9415
phi15 T7	phi15 67PfluR64PP	5,22	72,51	-139,23	149,68	0,9428
T7 Pf-10	PPPL-1 phi15	5,22	72,51	-139,23	149,67	0,9428
phi15 67PfluR64PP	phi15 Pf-10	5,11	72,51	-139,35	149,56	0,9441
phi15 67PfluR64PP	phi15 PPPL-1	5,07	72,51	-139,38	149,52	0,9444
PPPL-1 67PfluR64PP	phi15 Pf-10	4,99	72,51	-139,47	149,44	0,9454
PPPL-1 67PfluR64PP	phi15 PPPL-1	4,95	72,51	-139,50	149,41	0,9457
67PfluR64PP Pf-10	PPPL-1 Pf-10	4,93	72,51	-139,52	149,38	0,946
phi15 T7	67PfluR64PP phi15	4,88	72,51	-139,57	149,33	0,9465

Promoter-RNAP 1	Promoter-RNAP 2	Difference	Std Err Dif	Lower CL	Upper CL	p-Value
Pf-10 67PfluR64PP	T7 Pf-10	4,88	72,51	-139,58	149,33	0,9466
Pf-10 T7	67PfluR64PP PPPL-1	4,59	72,51	-139,86	149,04	0,9497
phi15 T7	Pf-10 T7	3,96	72,51	-140,49	148,41	0,9566
T7 PPPL-1	Pf-10 PPPL-1	3,92	72,51	-140,53	148,37	0,957
Pf-10 67PfluR64PP	67PfluR64PP 67PfluR64PP	3,72	72,51	-140,74	148,17	0,9593
67PfluR64PP phi15	67PfluR64PP PPPL-1	3,66	72,51	-140,79	148,12	0,9598
PPPL-1 T7	Pf-10 67PfluR64PP	3,59	72,51	-140,87	148,04	0,9607
Pf-10 phi15	Pf-10 67PfluR64PP	3,49	72,51	-140,97	147,94	0,9618
PPPL-1 phi15	phi15 T7	3,35	72,51	-141,10	147,80	0,9632
phi15 67PfluR64PP	67PfluR64PP PPPL-1	3,32	72,51	-141,13	147,77	0,9636
PPPL-1 Pf-10	PPPL-1 67PfluR64PP	3,27	72,51	-141,18	147,72	0,9641
Pf-10 67PfluR64PP	T7 67PfluR64PP	3,27	72,51	-141,18	147,72	0,9641
PPPL-1 67PfluR64PP	67PfluR64PP PPPL-1	3,20	72,51	-141,25	147,66	0,9649
PPPL-1 Pf-10	phi15 67PfluR64PP	3,15	72,51	-141,30	147,61	0,9654
67PfluR64PP Pf-10	phi15 T7	2,86	72,51	-141,59	147,31	0,9686
PPPL-1 Pf-10	67PfluR64PP phi15	2,81	72,51	-141,64	147,26	0,9692
phi15 T7	PPPL-1 Pf-10	2,07	72,51	-142,38	146,52	0,9773
PPPL-1 Pf-10	Pf-10 T7	1,89	72,51	-142,56	146,34	0,9793
67PfluR64PP T7	T7 PPPL-1	1,86	72,51	-142,59	146,31	0,9796
67PfluR64PP PPPL-1	phi15 Pf-10	1,78	72,51	-142,67	146,23	0,9805
67PfluR64PP PPPL-1	phi15 PPPL-1	1,75	72,51	-142,70	146,20	0,9808
T7 67PfluR64PP	T7 Pf-10	1,60	72,51	-142,85	146,06	0,9824
Pf-10 T7	PPPL-1 67PfluR64PP	1,39	72,51	-143,07	145,84	0,9848
Pf-10 T7	phi15 67PfluR64PP	1,27	72,51	-143,18	145,72	0,9861
67PfluR64PP 67PfluR64PP	T7 Pf-10	1,16	72,51	-143,29	145,61	0,9873
Pf-10 T7	67PfluR64PP phi15	0,93	72,51	-143,53	145,38	0,9899
PPPL-1 phi15	67PfluR64PP Pf-10	0,49	72,51	-143,96	144,94	0,9946
67PfluR64PP phi15	PPPL-1 67PfluR64PP	0,46	72,51	-143,99	144,91	0,995
T7 67PfluR64PP	67PfluR64PP 67PfluR64PP	0,44	72,51	-144,01	144,90	0,9951
67PfluR64PP phi15	phi15 67PfluR64PP	0,34	72,51	-144,11	144,79	0,9963

Promoter-RNAP 1	Promoter-RNAP 2	Difference	Std Err Dif	Lower CL	Upper CL	p-Value
phi15 67PfluR64PP	PPPL-1 67PfluR64PP	0,12	72,51	-144,33	144,57	0,9987
PPPL-1 T7	Pf-10 phi15	0,10	72,51	-144,35	144,55	0,9989
phi15 PPPL-1	phi15 Pf-10	0,03	72,51	-144,42	144,48	0,9996

Table S4: Connecting letters report of a pairwise Student's t-test of the cross-recognition assay between phage promoters and RNAPs. Levels not connected by same letter are significantly different.

Promoter	RNAP						Mean
T7	T7	A					2060,566
phi15	phi15		B				660,3784
PPPL-1	PPPL-1			C			245,7189
Pf-10	Pf-10			C	D		189,0884
T7	phi15				D	E	85,7572
67PfluR64PP	T7					E	41,8903
T7	PPPL-1					E	40,0312
Pf-10	PPPL-1					E	36,1123
PPPL-1	T7					E	29,5002
Pf-10	phi15					E	29,4009
Pf-10	67PfluR64PP					E	25,9146
T7	67PfluR64PP					E	22,6429
67PfluR64PP	67PfluR64PP					E	22,1996
T7	Pf-10					E	21,0388
PPPL-1	phi15					E	15,8159
67PfluR64PP	Pf-10					E	15,3239
phi15	T7					E	12,4631
PPPL-1	Pf-10					E	10,3922
Pf-10	T7					E	8,5055
67PfluR64PP	phi15					E	7,5807
phi15	67PfluR64PP					E	7,239
PPPL-1	67PfluR64PP					E	7,1203
67PfluR64PP	PPPL-1					E	3,9164
phi15	PPPL-1					E	2,1663
phi15	Pf-10					E	2,134
